# The HSP40 family chaperone isoform DNAJB6b prevents neuronal cells from tau aggregation

**DOI:** 10.1186/s12915-023-01798-6

**Published:** 2023-12-18

**Authors:** Ya-Lan Chang, Chan-Chih Yang, Yun-Yu Huang, Yi-An Chen, Chia-Wei Yang, Chia-Yu Liao, Hsun Li, Ching-Shyi Wu, Chin-Hsien Lin, Shu-Chun Teng

**Affiliations:** 1 Department of Microbiology, College of Medicine, National Taiwan University, No. 1, Section 1, Jen-Ai Road, Taipei, 10051 Taiwan; 2 Department of Neurology, National Taiwan University Hospital, No. 7, Chung-Shan South Road, Taipei, 10051 Taiwan; 3 Department of Pharmacology, College of Medicine, National Taiwan University, Taipei, 10051 Taiwan; 4 Center of Precision Medicine, National Taiwan University, Taipei, 10051 Taiwan

**Keywords:** Tau, Cochaperone, J-domain proteins, DNAJB6b, Alzheimer’s disease

## Abstract

**Background:**

Alzheimer’s disease (AD) is the most common neurodegenerative disorder with clinical presentations of progressive cognitive and memory deterioration. The pathologic hallmarks of AD include tau neurofibrillary tangles and amyloid plaque depositions in the hippocampus and associated neocortex. The neuronal aggregated tau observed in AD cells suggests that the protein folding problem is a major cause of AD. J-domain-containing proteins (JDPs) are the largest family of cochaperones, which play a vital role in specifying and directing HSP70 chaperone functions. JDPs bind substrates and deliver them to HSP70. The association of JDP and HSP70 opens the substrate-binding domain of HSP70 to help the loading of the clients. However, in the initial HSP70 cycle, which JDP delivers tau to the HSP70 system in neuronal cells remains unclear.

**Results:**

We screened the requirement of a diverse panel of JDPs for preventing tau aggregation in the human neuroblastoma cell line SH-SY5Y by a filter retardation method. Interestingly, knockdown of DNAJB6, one of the JDPs, displayed tau aggregation and overexpression of DNAJB6b, one of the isoforms generated from the DNAJB6 gene by alternative splicing, reduced tau aggregation. Further, the tau bimolecular fluorescence complementation assay confirmed the DNAJB6b-dependent tau clearance. The co-immunoprecipitation and the proximity ligation assay demonstrated the protein–protein interaction between tau and the chaperone–cochaperone complex. The J-domain of DNAJB6b was critical for preventing tau aggregation. Moreover, reduced DNAJB6 expression and increased tau aggregation were detected in an age-dependent manner in immunohistochemical analysis of the hippocampus tissues of a mouse model of tau pathology.

**Conclusions:**

In summary, downregulation of DNAJB6b increases the insoluble form of tau, while overexpression of DNAJB6b reduces tau aggregation. Moreover, DNAJB6b associates with tau. Therefore, this study reveals that DNAJB6b is a direct sensor for its client tau in the HSP70 folding system in neuronal cells, thus helping to prevent AD.

**Supplementary Information:**

The online version contains supplementary material available at 10.1186/s12915-023-01798-6.

## Background

By the year 2050, an estimated 22% of the global population will be over 60 years of age [1]. As society ages, the number of patients suffering from neurodegenerative disorders increases dramatically. AD is the most common neurodegenerative disorder with clinical presentations of progressive cognitive decline and memory deterioration. The pathology hallmarks of AD are neuronal phospho-tau accumulation as neurofibrillary tangles and extra-neuronal amyloid plaque deposition in the hippocampus, causing progressive neuronal degeneration and associated memory decline. Neuronal loss is largely parallel with tau neurofibrillary tangle formation [2].

Tau protein is a major protein to maintain microtubule stability [3–5]. During the disease process of AD, tau is hyperphosphorylated and accumulates as neurofibrillary tangles, which contribute to AD pathology [6, 7]. Accumulation of tau in neurons disturbs microtubules, proper axonal transport, and mitochondrial function, leading to neuronal death [8]. In addition to the passive leakage caused by neuronal death, a pre-synaptic mechanism stimulated by the neuronal activity was also proposed to release the pathological tau leading to spreading to different brain regions through the process of “prion-like propagation” [9, 10]. Consistent with these hypotheses, the progression of disease pathology of AD was proposed by Braak’s staging, classified from groups I to VI, starting at stage I with an accumulation of granular tau oligomers in the frontal cortex [11]. Similar aggregated proteins are also observed in many neurodegenerative disorders [12]. Neuronal α-synuclein aggregates are found in Parkinson’s disease [13]. TDP43 is accumulated in amyotrophic lateral sclerosis and frontotemporal dementia [14]. All these observations indicate that the protein folding problem should be a major fundamental cause for the formation of neurodegenerative disorders.

Cells have several mechanisms to prevent tau protein aggregations, including chaperones, cochaperones, ubiquitin–proteasome degradation system, aggresomes to stabilize the aggregates, and lysosomal degradation pathway by a process called chaperone-mediated autophagy [15, 16]. The first molecular chaperone was found by Sternberg in 1973 in studies of mutations that disrupted bacteriophage λ head formation [17]. Later, Boulon et al. discovered that the heat shock protein (HSP) HSP70 system is the major chaperone machinery for cellular protein folding [18]. In addition to chaperones, the complicity and specificity of protein folding machines largely depend on cochaperones [19]. Active participation of cochaperone proteins at various stages of the chaperone folding cycle is crucial for the completion of the folding process [20]. Cochaperones can bind to specific domains of HSP to stabilize its conformation and further modulate its function. Besides, cochaperones can recruit specific clients for the folding system [21]. Therefore, both HSP70 and cochaperones control folding and proteostasis.

JDPs are a family of cochaperone proteins that recruit specific clients/substrates to the HSP70 protein folding system [19]. They protect cells from all kinds of stresses in different cellular backgrounds. DnaJ was first identified in *Escherichia coli*. It contains a J-domain to bind and stimulate the ATPase activity of the bacterial Hsp70/DnaK [22]. Genes that encode proteins homologous to *E. coli* DnaJ were further identified in organisms ranging from yeast to plants and humans. In a folding cycle, JDPs interact with substrates and deliver newly translated peptides or unfolded substrates/clients to HSP70, which prevents further misfolding and aggregation and facilitates refolding. There are over 50 JDPs in humans and they have been grouped into three types based on their structures [23]. The J-domain is located at the N terminus of the A- and B-type JDPs, while the J-domain is in the middle of the C-type JDPs. Different JDPs provide specificity for assembling various JDP-HSP70 chaperone machinery while carrying discriminatory phylogenetic signatures that specify their function. For example, yeast Sis1, a B-type JDP, recruits more Hsp70 to the aggregate and drives polypeptide disentanglement, while the binding of Ydj1, an A-type JDP, favors the reactivation of solubilized misfolding substrates [24]. JDPs also bind and promote the ATPase activity of HSP70 [22], whereas several JDPs such as *E. coli* DnaJ and yeast Ydj1 can maintain soluble clients from aggregation by themselves through ATP-independent association with the substrates [25].

Many studies have shown that JDPs are involved in the remodeling of neurodegenerative disease-related proteins [26, 27]. Overexpression of DNAJA1 results in a decrease of tau protein in HeLa cells [28]. An in vitro large-interaction analysis showed DNAJA2 as a suppressor of tau aggregation [29]. Two class B JDPs, DNAJB1 and DNAJB4, were identified to enable HSC70 to disaggregate tau fibrils in vitro [30]. Recently, knockout of DNAJC7 was found to decrease the aggregate clearance and increase intracellular tau seeding in HEK293 cells [31]. However, there was no systematic approach to functionally screen for the roles of JDPs in AD in human neuronal cells. To understand how human neuronal cells recognize and resolve tau aggregation and how tau is aggregated in neurons in the progress of AD, we examined whether knockdown of JDPs may change the aggregation of tau. Among over 50 human JDPs [23], we screened 11 JDPs that are highly expressed in neuronal cells and/or are related to neurodegenerative diseases [27]. Interestingly, only DNAJB6 knockdown cells displayed tau aggregation. DNAJB6 is expressed as two alternative splicing isoforms, DNAJB6a (36 kDa) and DNAJB6b (27 kDa) [32]. Overexpression of the DNAJB6b isoform reduced tau aggregation. We examined the requirement of the J-domain in DNAJB6b-mediated tau recruitment. The relationship between DNAJB6 and tau was also investigated in a mouse model of tau pathology.

## Results

### DNAJB6 decreases tau aggregation in human neuroblastoma cells

**Fig. 1:**
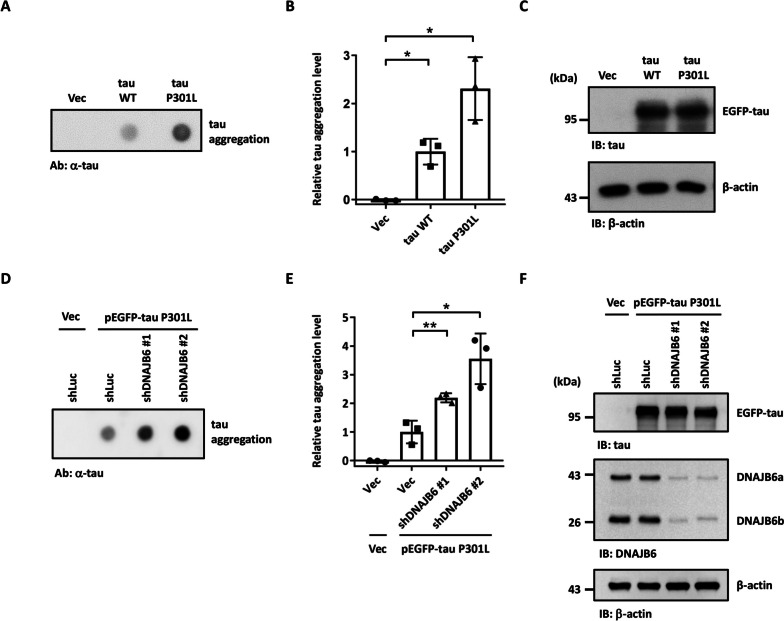
DNAJB6 knockdown increases tau aggregation. **A** The filter trap assay detected tau aggregation. The cellular lysate of SH-SY5Y neuroblastoma cells transfected with the empty vector (pEGFP-C1), wild-type tau, or mutated tau P301L for 48 h was filtered through cellulose acetate membranes. The dot blot assay was then performed with a tau antibody to detect trapped tau. **B** Quantification of tau aggregates. The values were given as mean ± standard deviation (S.D.) (*n* = 3, *P < 0.05, unpaired two-tailed Welch’s *t*-test). **C** tau expression level of cells in **A** was confirmed by immunoblotting. β-actin was used as a loading control for the normalization in **B**. **D** shLuc or shDNAJB6 SH-SY5Y cells were transfected with an empty vector or tau P301L for 48 h. The cellular lysate was filtered through cellulose acetate membranes and retained proteins were stained with a tau antibody. **E** Quantification of tau aggregates. The values were given as mean ± S.D. (*n* = 3, *P < 0.05, **P < 0.01, unpaired two-tailed Student’s *t*-test). **F** Expression of tau and knockdown of DNAJB6 in cells shown in **D** were determined by immunoblotting using tau and DNAJB6 antibodies, respectively. β-actin was used as a loading control for the normalization in **E**. The individual data values of the replicates in **B** and **E** are listed in Additional file 2

**Fig. 2:**
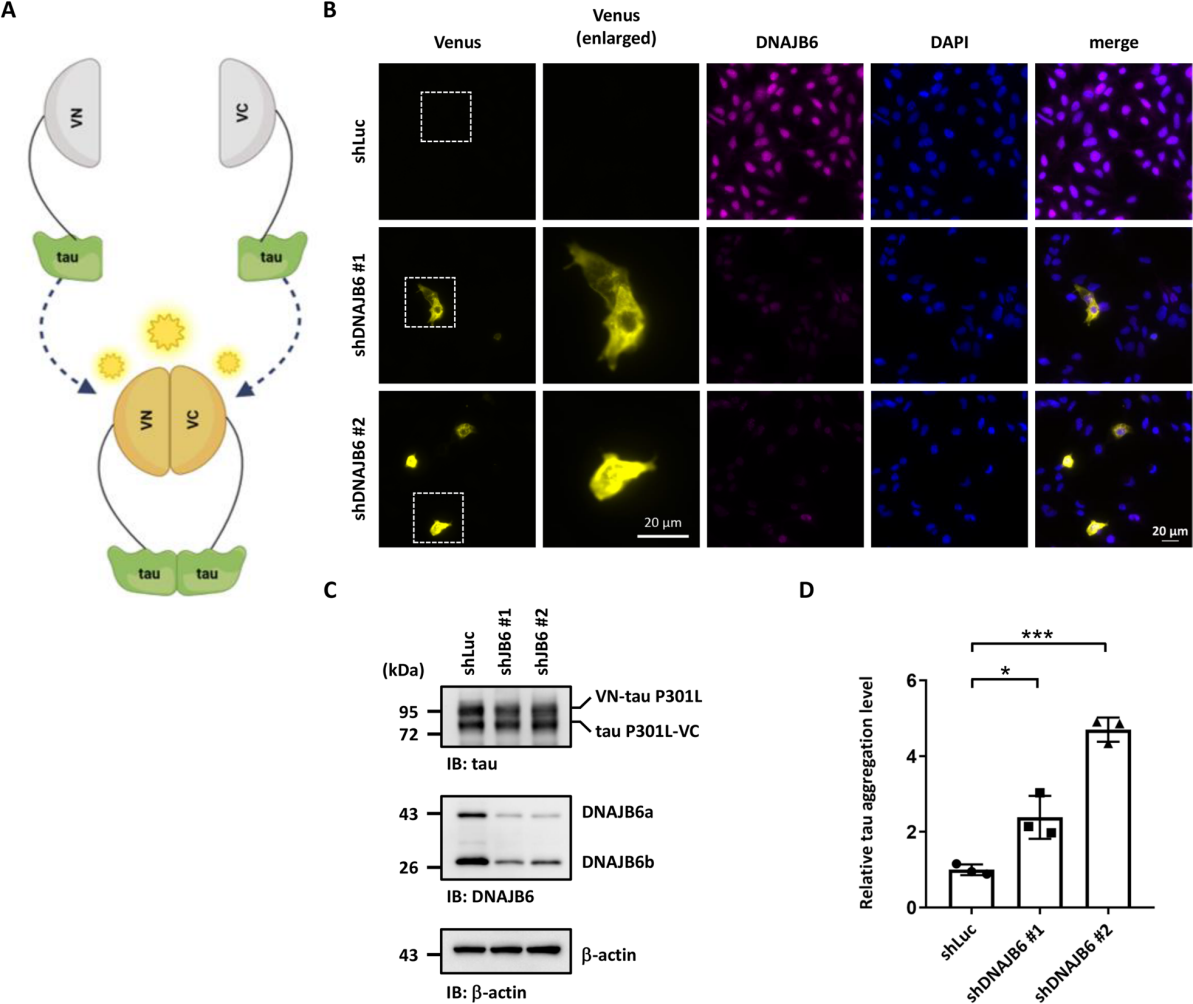
Visualization of DNAJB6-dependent tau aggregation in neuroblastoma cells. **A** Schematic representation of BiFC maturation upon tau aggregation. Tau P301L was fused to one of the halves of the Venus protein (VN- or VC-). Aggregation of tau protein promotes the complementation of two non-fluorescent Venus halves and leads to the emission of fluorescence. **B** Representative fluorescent images of BiFC and DNAJB6. shLuc or shDNAJB6 SH-SY5Y cells was co-transfected with the BiFC-tau P301L constructs. The fluorescence emitted by Venus complementation was recorded 48 h after transfection. Cells were immunostained with a DNAJB6 antibody. Nuclei were counterstained with DAPI. Enlarged Venus images of the representative cells corresponding to the selected regions are shown. Scale bar: 20 μm. **C** Immunoblotting with anti-tau antibodies indicated the expression level of BiFC-tau P301L in SH-SY5Y cells. The immunoblotting of anti-DNAJB6 antibodies determined the knockdown of DNAJB6. **D** Quantification of BiFC-fluorescence of **B**. The percentage of fluorescent (BiFC positive) SH-SY5Y cells to the total number of cells was quantified. The values were given as mean ± S.D. (*n* = 3, *P < 0.05, ***P < 0.001, unpaired two-tailed Student’s *t*-test). The individual data values of the replicates in **D** are listed in Additional file 2

To understand how tau is aggregated in neurons in the process of AD, we screened JDPs that may be used to resolve the tau aggregation. We examined whether knockdown of JDPs may change the aggregation of tau. We first established an assay system to follow protein aggregation (Fig. 1A-C). The dot blot assay, a filter retardation method to capture protein aggregates in a cellulose acetate membrane, has been used to detect and quantify protein aggregation in human neuroblastoma cell line SH-SY5Y [33]. Cells expressing the disease-associated tau with a proline to leucine mutation at residue 301 (tau P301L) showed higher tau aggregation levels compared to cells expressing normal tau protein (Fig. 1A-C). Moreover, the cellular Triton X-100 insoluble fractionation assay [34, 35] also showed that cells expressing the tau P301L mutant exhibited a significant increment of the insoluble form of tau (Additional file 1: Fig. S1) [36]. We then transfected the disease-associated P301L mutation tau and tested tau aggregation in the JDP-knockdown cells. The JDP’s knockdown efficiency was analyzed on mRNA or protein level using quantitative reverse transcription PCR (qRT-PCR) or immunoblotting, respectively (Additional file 1: Fig. S2A-K). Using this dot blot approach, we screened 11 JDPs that have been shown to have mutations or potential roles in neurodegenerative diseases, including DNAJA1, DNAJA2, DNAJA3, DNAJB1, DNAJB5, DNAJB6, DNAJC5, DNAJC6, DNAJC11, DNAJC13, and DNAJC29 [27, 37]. DNAJB2 and DNAJC19 were not on the screen due to the knockdown failure. We found that knockdown of DNAJA3 decreased tau aggregation, while DNAJB6 knockdown cells displayed increased tau aggregation (Fig. 1D-F, Additional file 1: Fig. S3A-C). DNAJB6 was previously identified to resolve polyQ_119_ aggregation [38]. To understand whether DNAJB6 can also decrease the aggregation of another toxic protein, SH-SY5Y cells were treated with or without rotenone, an electron transport chain inhibitor that can induce α-synuclein aggregation [33, 39, 40]. Knockdown of DNAJB6 did not lead to α-synuclein aggregation under rotenone treatment (Additional file 1: Fig. S4A-C). To further confirm the role of DNAJB6 in regulating tau aggregation, a tau bimolecular fluorescence complementation (BiFC) assay was used to investigate tau aggregation in DNAJB6 knockdown cells [41]. Non-fluorescent N- and C-terminal compartments of Venus protein are fused to disease-associated full-length tau P301L and co-expressed in a cell. Only when tau assembles, the Venus fluorescence turns on as an indication of tau aggregation (Fig. 2A). Compared to the knockdown of firefly luciferase (shLuc) control cells, knockdown of DNAJB6 resulted in more tau-BiFC positive signals, indicating a higher level of tau aggregation in the cells (Fig. 2B-D). Moreover, knockdown of DNAJB6 also further increased the insoluble fractionation of tau (Additional file 1: Fig. S5A and S5D). To investigate whether the increased tau protein aggregation was caused by alteration of the levels of heat shock proteins, the protein levels of a subset of HSP70s, HSPA8, HSPA1A, HSPA9, and BiP were detected by immunoblotting in shLuc and shDNAJB6 SH-SY5Y cells. Knockdown of DNAJB6 did not change the protein levels of these HSP70 molecular chaperones (Additional file 1: Fig. S6). Together, these results suggest that DNAJB6 may untangle tau aggregation in human neuroblastoma cells.

Apoptosis is the central mechanism of cell death driven by a pathological tau protein in cellular models [42–44]. A previous study showed that expression of the toxic truncated tau protein activates a caspase-3-independent caspase-9-mediated cell death [45]. To investigate the effect of DNAJB6 on cellular death driven by tau aggregation, the protein levels of the cellular apoptotic marker, cleaved 37 kDa caspase-9, was detected in shLuc or shDNAJB6 SH-SY5Y cells transfected with tau P301L mutant. Interestingly, overexpression of the P301L mutant slightly induced the caspase-9 apoptosis pathway, and the shDNAJB6 cells displayed a stronger induction of caspase-9 (Additional file 1: Fig. S7). These results indicate that overexpression of the tau P301L mutant and loss of DNAJB6 can induce the caspase-9-mediated apoptosis pathway.

### DNAJB6b is the major isoform to decrease tau aggregation

**Fig. 3:**
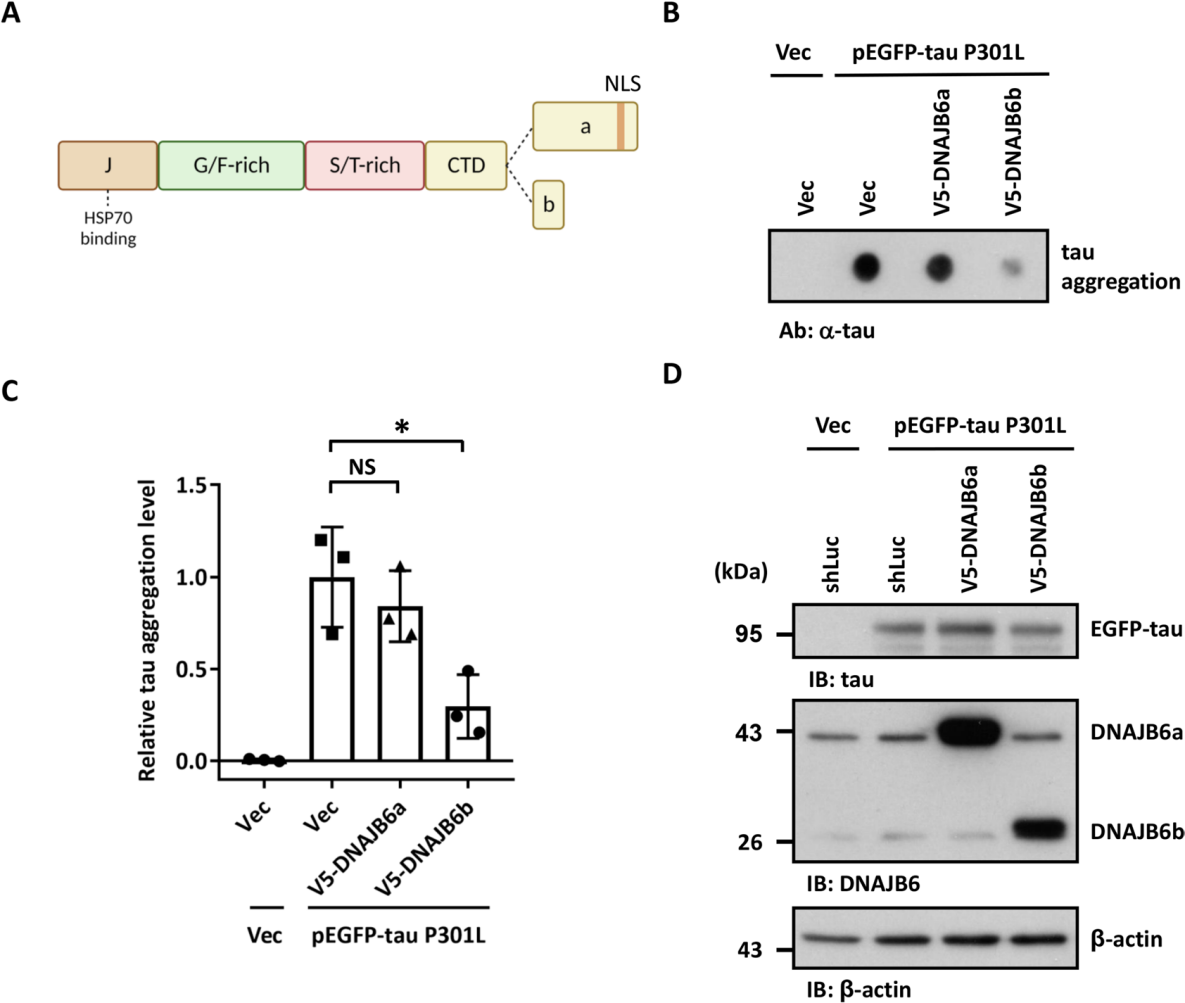
Overexpression of DNAJB6 isoform b reduces tau aggregation. **A** A diagram of various domains of DNAJB6 protein. The alternatively spliced C-terminal parts of isoforms a and b are indicated. DNAJB6a and DNAJB6b share the N-terminal 231 amino acids but differ in their C-terminal region and their cellular localization. DNAJB6a, the longer isoform, contains an NLS and predominantly localizes in the nucleus, whereas the shorter DNAJB6b primarily localizes in the cytoplasm. **B** The cellular lysate of SH-SY5Y cells which co-transfected an empty vector or mutated tau P301L together with an empty vector (pcDNA/FRT/TO-V5), V5-DNAJB6a, or V5-DNAJB6b for 48 h was filtered through cellulose acetate membranes. Retained proteins were stained with a tau antibody. **C** Quantification of aggregation tau. The values were given as mean ± S.D. (*n* = 3, *P < 0.05, N.S. non-significant, unpaired two-tailed Student’s *t*-test). The individual data values of the replicates in **C** are listed in Additional file 2. **D** The expression levels of tau and DNAJB6 were determined by immunoblotting using tau and DNAJB6 antibodies, respectively. β- actin was used as a loading control for the normalization in **C**

**Fig. 4:**
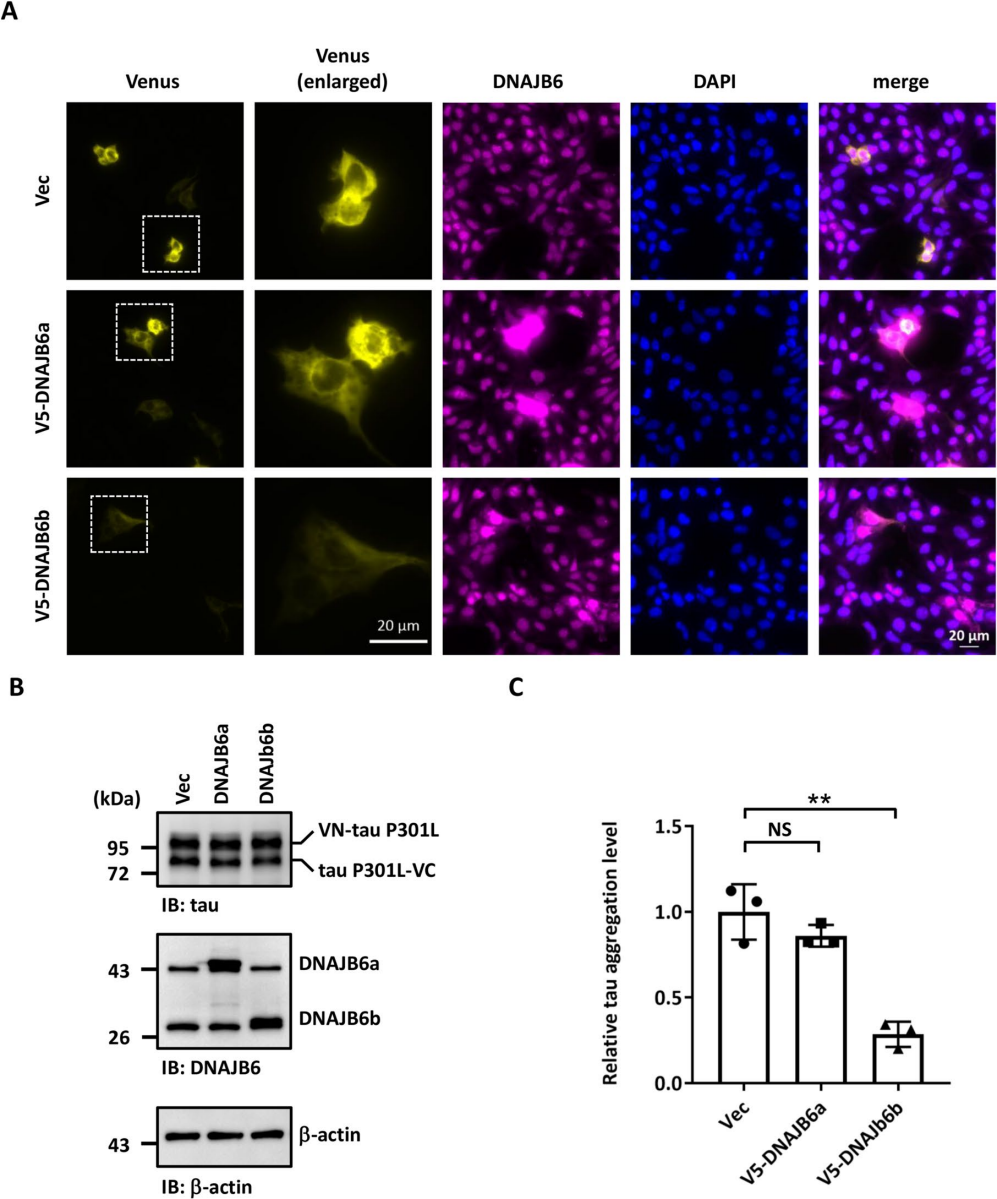
Expression of DNAJB6b decreases visualized tau aggregation in neuroblastoma cells. **A** Representative fluorescent images of BiFC and DNAJB6 in SH-SY5Y cells that co-expressed the BiFC-tau P301L constructs and an empty vector, V5-DNAJB6a, or V5-DNAJB6b. The fluorescence emitted by Venus complementation was imaged 48 h after transfection. Cells were immunostained with DNAJB6 and counterstained with DAPI. Enlarged Venus images of the representative cells corresponding to the selected regions are shown. Scale bar: 20 μm. **B** The expression levels of BiFC-tau P301L and DNAJB6 were indicated by immunoblotting with tau and DNAJB6 antibodies in SH-SY5Y cells, respectively. **C** Quantification of BiFC-fluorescence of **A**. The percentage of BiFC-positive SH-SY5Y cells to the total number of cells was quantified. The values were given as mean ± S.D. (*n* = 3, **P < 0.01, unpaired two-tailed Student’s *t*-test). The individual data values of the replicates in **C** are listed in Additional file 2

Like other members of DNAJ proteins, DNAJB6 contains 4 domains, including an N-terminal J-domain that contains a conserved histidine-proline-aspartate (HPD) motif, which is required for the protein–protein interaction with HSP70 and stimulation of its ATPase activity [19, 46]. Followed by the J-domain is a glycine and phenylalanine-rich (G/F) region, containing most disease-related mutations [47, 48]. The serine/threonine-rich (S/T) region is located in a likely client-binding cleft [49–51] (Fig. 3A). DNAJB6 is expressed as two alternative splicing isoforms, DNAJB6a and DNAJB6b (Fig. 3A) [32]. The long isoform DNAJB6a contains a nuclear localization signal (NLS) in its C-terminal domain and is majorly located in the nucleus, while the short isoform DNAJB6b is predominantly located in the cytoplasm and transiently accumulates to nuclei upon heat shock and hypoxia [47]. To understand which isoform is critical for tau aggregation, we overexpressed DNAJB6a and DNAJB6b in SH-SY5Y cells. Overexpression of DNAJB6a or DNAJB6b did not change the protein levels of the molecular chaperones HSPA8, HSPA1A, HSPA9, and BiP (Additional file 1: Fig. S8). Interestingly, overexpression of DNAJB6b, but not DNAJB6a, reduced the insoluble form of tau and prevented its aggregation (Fig. 3B-D and Additional file 1: Fig. S5B and S5E). Furthermore, the tau-BiFC assay showed that DNAJB6b displayed a stronger impact on decreasing tau aggregation in SH-SY5Y cells (Fig. 4A-C). The cellular Triton X-100 insoluble fractionation assay also demonstrated that DNAJB6b expression reduced tau aggregation in the insoluble fraction (Additional file 1: Fig. S5B and S5E). These results indicate that DNAJB6b is the major JDP decreasing tau aggregation in human neuroblastoma cells.

### DNAJB6 interacts with cytoplasmic HSP70 and tau

**Fig. 5:**
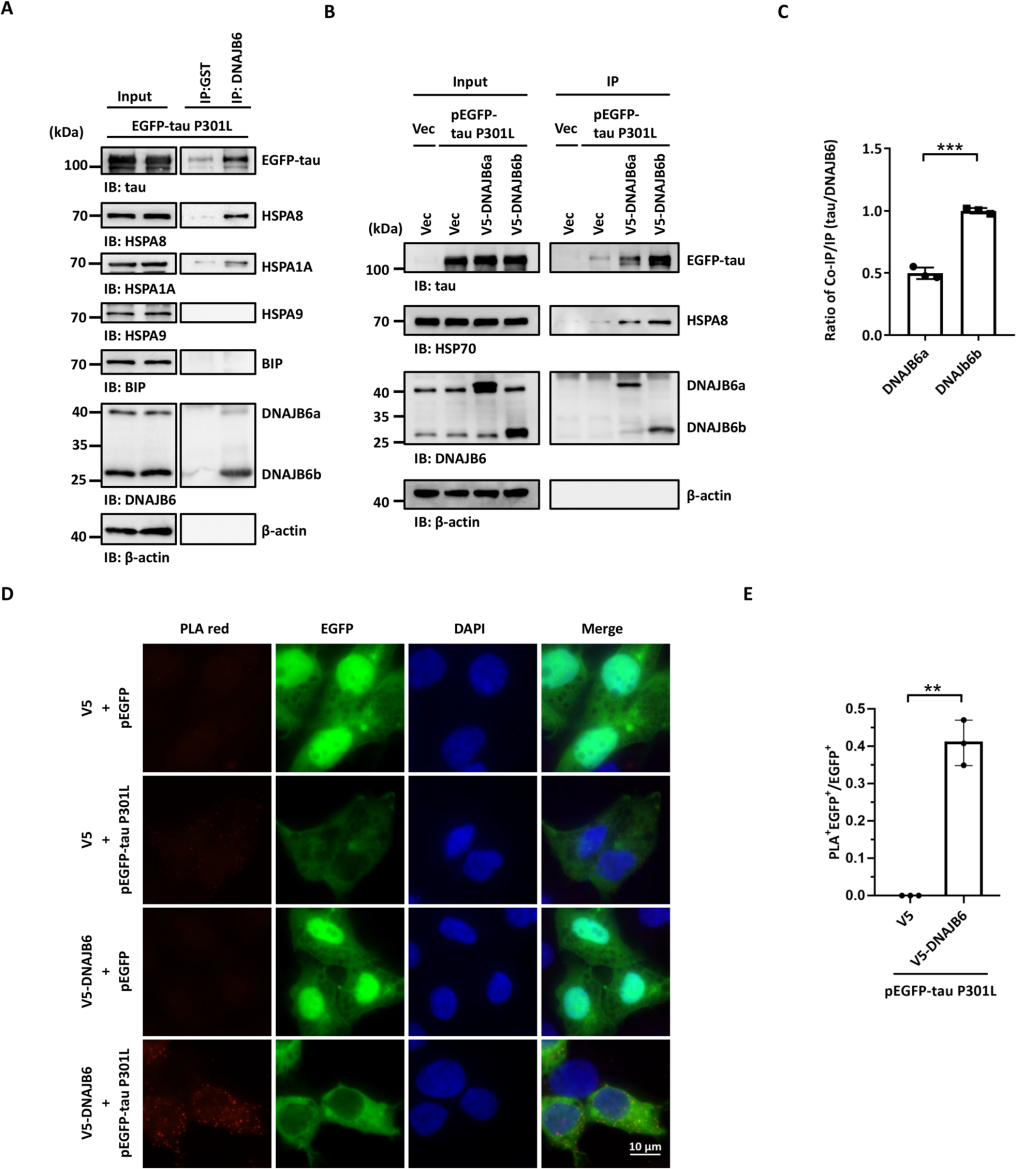
DNAJB6 is associated with tau and HSP70. **A** Co-immunoprecipitation assay indicated that DNAJB6 is associated with tau and HSPA8. SH-SY5Y cells expressing tau P301L were immunoprecipitated using an anti-DNAJB6 antibody to pull down endogenous DNAJB6. An anti-GST antibody served as a negative control. Precipitated and co-precipitated proteins were analyzed by immunoblotting using indicated antibodies. The images are representatives of three biological replicates.** B** SH-SY5Y cells co-expressing tau P301L and an empty vector, V5-DNAJB6a, or V5-DNAJB6b for 48 h were immunoprecipitated using an anti-V5 antibody to pull down V5-DNAJB6a or V5-DNAJB6b. Co-immunoprecipitated proteins were detected as described in **A**.** C** Quantification of the ratio of co-immunoprecipitated tau to immunoprecipitated DNAJB6a or DNAJB6b in **B**. The values were given as mean ± S.D. (*n* = 3, ***P < 0.001, unpaired two-tailed Student’s *t*-test). **D** V5-DNAJB6b and EGFP-tau plasmids were co-transfected into SH-SY5Y cells. 24 h post-transfection, cells were seeded on slides for an additional 24 h. Cells seeded on slides were then hybridized with tau and V5 primary antibodies to detect tau and DNAJB6b, respectively. When tau and DNAJB6 interact, the PLA probes on secondary antibodies were ligated and amplified. The red PLA signals showing protein interaction were detected by fluorescent microscopy. Scale bar: 10 μm. **E** Quantification of red PLA signal of **D**. The percentage of PLA-positive SH-SY5Y cells to the tau P301L transfected cells (EGFP-positive) was quantified. The values were given as mean ± S.D. (*n* = 3, **P < 0.01, unpaired two-tailed Welch’s *t*-test). The individual data values of the replicates in **C** and **E** are listed in Additional file 2

DNAJB6 interacts with HSP70 and stimulates heat shock protein ATPase activity through its J-domain. There are several human HSP70 proteins, including the stress-inducible HSPA1A, organelle-specific BiP and HSPA9, and the constitutively expressed isoform HSPA8 [52, 53]. The co-immunoprecipitation of endogenous DNAJB6 demonstrated that DNAJB6 associates with HSPA8 and HSPA1A**,** but not the endoplasmic reticulum (ER)-specific isoform BiP, and mitochondria-specific isoform HSPA9 (Fig. 5A). Since we found that DNAJB6 can decrease tau aggregation in human neuroblastoma cells, we would like to know whether tau, the client, is associated with HSPA8-DNAJB6, the chaperone–cochaperone complex. The co-immunoprecipitation of endogenous DNAJB6 demonstrated the association between DNAJB6 and tau (Fig. 5A). To determine which isoform interacts with tau, the V5-tagged DNAJB6a or DNAJB6b was co-expressed with tau P301L into SH-SY5Y cells. Co-immunoprecipitation results showed that both a and b forms of DNAJB6 could interact with tau in vitro while the b form exhibited higher binding ability with tau (Fig. 5B-C). We further performed in situ protein–protein interaction (at distances < 40 nm) of DNAJB6b and tau by the proximity ligation assay (PLA) [54, 55]. SH-SY5Y cells co-overexpressing pEGFP-tau P301L and V5-DNAJB6b exhibited a PLA positive signal (Fig. 5D-E), indicating the interaction of DNAJB6b and its client, tau. These results suggest that DNAJB6b is a cochaperone that interacts with tau and helps to untangle tau aggregations.

### DNAJB6b H31Q mutation loses its ability to interact with HSP70 but not its ability to carry tau protein

**Fig. 6:**
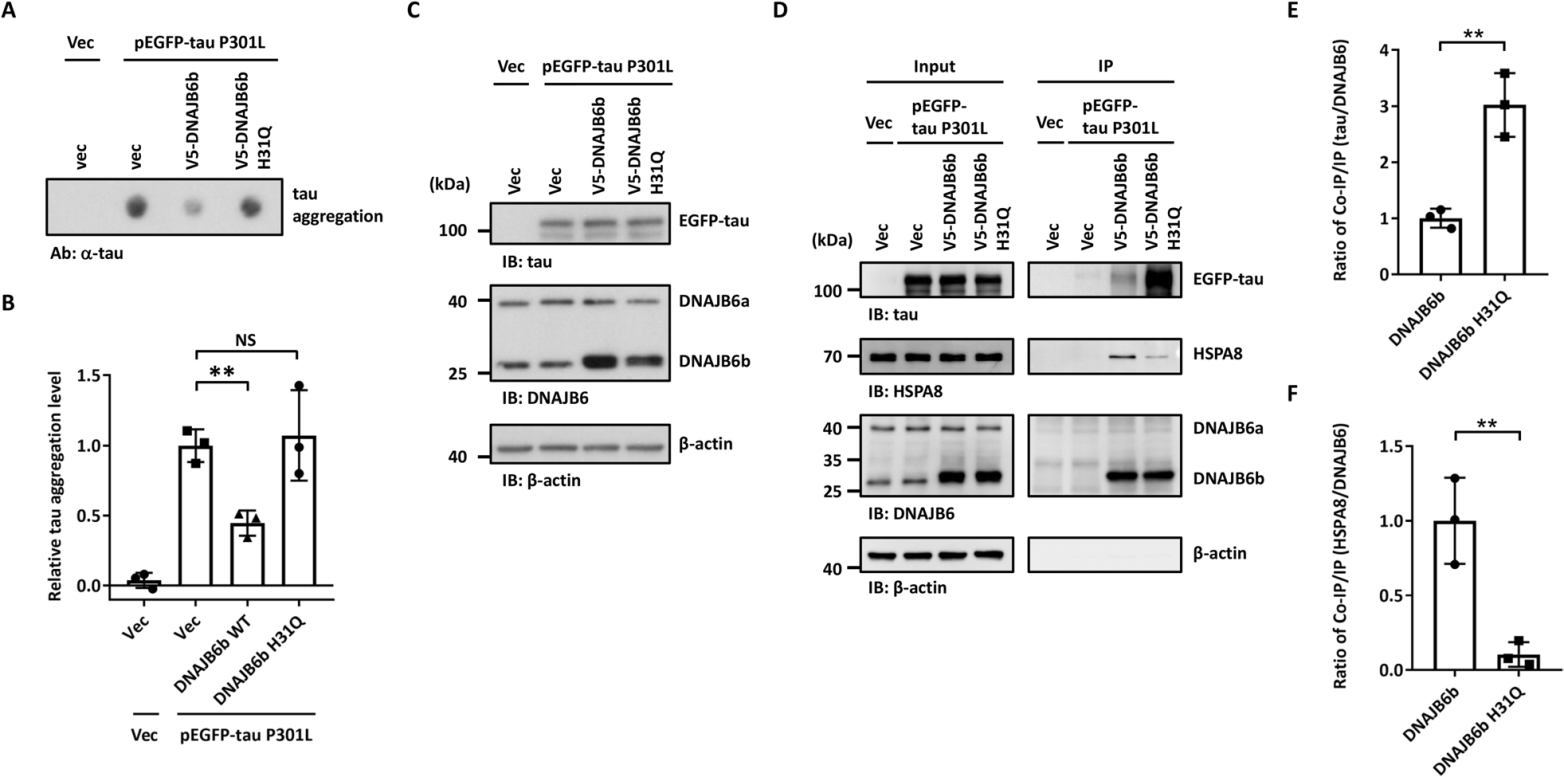
The J-domain of DNAJB6b is critical for preventing tau aggregation. **A** Cellular lysate of SH-SY5Y cells co-transfected with mutated tau P301L together with an empty vector, V5-DNAJB6b, or V5-DNAJB6b H31Q plasmids for 48 h were filtered through cellulose acetate membranes. The dot blot assay was then performed with a tau antibody to detect trapped tau. **B** Quantification of tau aggregates is shown in **A**. The values were given as mean ± S.D. (*n* = 3, **P < 0.01, unpaired two-tailed Student’s *t*-test). **C** The expression levels of tau and DNAJB6b were determined by immunoblotting using tau and DNAJB6 antibodies, respectively. β-actin was used as a loading control for the normalization in **B**.** D** SH-SY5Y cells co-expressing tau P301L and an empty vector, V5-DNAJB6b or V5-DNAJB6b H31Q for 48 h were immunoprecipitated using an anti-V5 antibody to pull down DNAJB6b. Precipitated and co-precipitated proteins were analyzed by immunoblotting using indicated antibodies. **E–F** Quantification of the ratio of co-immunoprecipitated tau (**E**) and HSPA8 (**F**) to immunoprecipitated DNAJB6b or DNAJB6b H31Q in (**D**). The values were given as mean ± S.D. (*n* = 3, **P < 0.01, unpaired two-tailed Student’s *t*-test). The individual data values of the replicates in **B**, **E**, and **F** are listed in Additional file 2

The JDPs usually function within the context of the HSP70 machinery. The HPD motif in the J domain of DNAJB6 is crucial for the interaction with HSP70 and for facilitating the ATPase activity of HSP70. To understand whether the J-domain of DNAJB6b is essential for preventing the aggregation of tau or not, the histidine residue in the conserved HPD motif of the J-domain of DNAJB6b was mutated to glutamate (H31Q) to inactivate the J-domain [56]. As expected, this mutant could not suppress tau aggregation in the cells, as the dot blot and Triton X-100 insoluble fractionation assays showed that the DNAJB6b H31Q mutant lost its ability to decrease tau aggregation (Fig. 6A-C and Additional file 1: Fig. S5C and S5F). We next asked whether the H31Q mutation could affect the interaction between DNAJB6 and tau or not. The V5-tagged wild-type or H31Q mutant DNAJB6b was co-expressed with tau P301L in SH-SY5Y cells, followed by co-immunoprecipitation. The H31Q mutation on DNAJB6b did not lose its association with tau but decreased the DNAJB6b-HSP70 interaction (Fig. 6D-F). These results imply that the DNAJB6b H31Q mutation loses its ability to interact with HSP70 and also its ability to suppress tau aggregation, but not its ability to bind to tau protein.

### DNAJB6 expression and tau phosphorylation are inversely correlated in an age-dependent manner in a mouse model of tau pathology

**Fig. 7:**
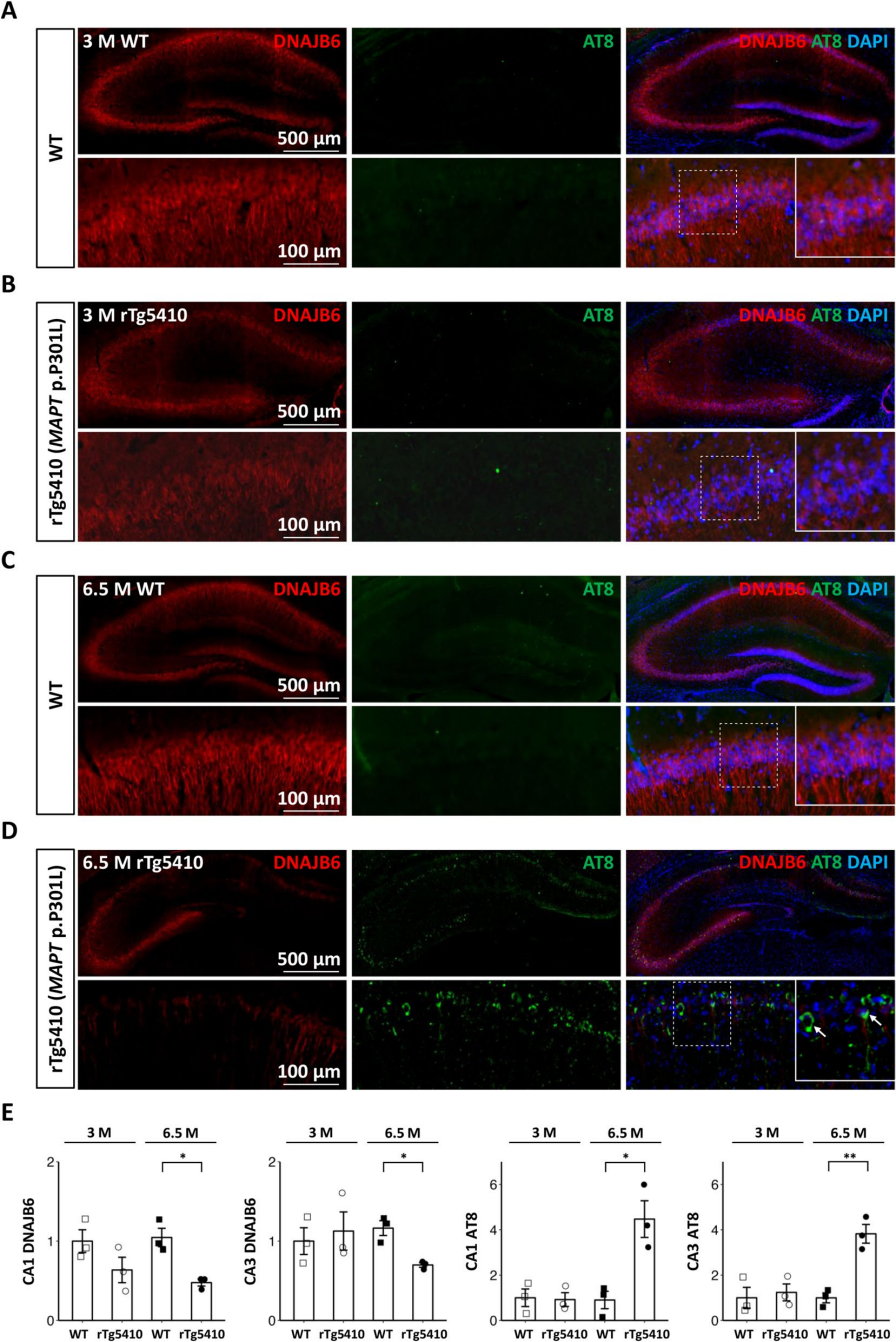
Increased tau aggregation correlates with reduced DNAJB6 expression in an age-dependent manner in a mouse model of tau pathology. **A–D** Immunohistochemistry staining was conducted in the coronal sections of the wild-type (**A** and **C**) and rTg4510 (**B** and **D**) mice. The coronal slices were at the level Bregma -2.06 mm. Representative images display the DNAJB6 (red), AT8 (green), and DAPI (blue) stainings throughout the hippocampal region (top) and in the CA1 and CA3 regions of the hippocampal neurons (bottom) in rTg4510 mice and non-transgenic littermate controls at the age of 3 (**A-B**) and 6.5 months (**C-D**). **E** Quantitative analysis of expression of DNAJB6 and AT8 in CA1 and CA3 regions of rTg4510 mice and littermate controls at the age of 3 and 6.5 months (*n* = 3, *P < 0.05, **P < 0.01, two-tailed Student’s *t*-test). The individual data values of the replicates in **E** are listed in Additional file 2

To validate whether the protective effects of untangling tau aggregations by DNAJB6 can be observed in mice, the correlation of DNAJB6 expression and phosphorylated tau, a cause of tau aggregation, was examined in a transgenic mouse model. The transgenic rTg4510 mouse expresses four-repeat human tau with the P301L mutation and has been shown to express approximately 13-fold higher tau, resulting in the development of hippocampal tau pathology associated with progressive cognitive decline [57]. We performed immunohistochemical analysis in the hippocampal region of the rTg4510 mice (Fig. 7). Hippocampus is one of the brain regions affected early on in AD, and its atrophy is significantly associated with memory loss and learning impairment. Pathologic tau occurs in a hyperphosphorylated state [58, 59]. The phosphorylation of neuronal tau at serine 202 and threonine 205 residues is the driving force for its aggregation and is recognized by the phosphorylation-specific monoclonal antibody, AT8 [60]. The phosphorylation of neuronal tau at serine 202 and threonine 205 residues was undetectable and the expression level of DNAJB6 was comparable in the hippocampal CA3 regions in rTg4510 mice and littermate controls at the age of 3 months (Fig. 7A-B, statistics in Fig. 7E). However, an increased phospho-tau associated with reduced DNAJB6 expression in the CA3 region of the hippocampal neurons was observed in the aged rTg4510 mice compared to littermate non-transgenic wild-type controls at the age of 6.5 months (Fig. 7C-D, statistics in Fig. 7E). These observations suggest an age-dependent progressive reduction of DNAJB6 expression accompanied by an increased expression of phospho-tau in the hippocampal neurons in a mouse model of tau pathology.

## Discussion

Due to population aging, the number of people globally affected by dementia is estimated to dramatically increase from 2020 to 2050 [61]. Although cellular tau aggregation is a major cause of AD, how tau aggregation is prevented in neurons is still unclear. Under pathological conditions, detached tau which loses the affinity to microtubules forms aggregates in the cytoplasm [62, 63]. The JDP-mediated cargo delivery should be the first and the rate-limiting step in the HSP70 system. In this study, we identify that overexpression of DNAJB6b, but not DNAJB6a, reduces the insoluble form of tau and prevents its aggregation. Both DNAJB6a and DNAJB6b can interact with tau in vitro; however, b form exhibits higher binding ability with tau. Tau is mainly found in the cytosol of neuronal cells. DNAJB6b predominantly localizes in the cytoplasm, whereas DNAJB6a containing an NLS is mainly delivered to the nucleus. The different subcellular localization of these proteins might be the reason for the different binding ability between a and b forms of DNAJB6 and tau. Our study demonstrates that the tau can be recognized majorly by DNAJB6b to help deliver it to the cytoplasmic HSP70 folding system, and we also show that tau can bind to DNAJB6b in situ. Therefore, we suggest that DNAJB6b is the major JDP used to recruit tau to the HSP70 folding system in human neuroblastoma cells. After DNAJB6b interacts with tau, the DNAJB6b-HSP70 machinery may disaggregate fibrils in aqueous conditions into smaller non-toxic species [64]. If DNAJB6b fails to execute its function, such as a J-domain mutation, it would cause tau to fail to be transmitted to the HSP70 folding cycle, resulting in continuous accumulation and aggregation of tau (Additional file 1: Fig. S9). In cells, molecular chaperone system HSP70 and its JDP cochaperones transiently interact with a myriad of client proteins [65]. However, the association of DNAJB6b H31Q with tau was dramatically increased compared to that of the wild-type DNAJB6b (Fig. 6D-E). This might indicate an unusually stable interaction when the JDPs lose their ability to hand off the clients to HSP70s for the following folding step.

DNAJB6 is a member of the HSP40 family of molecular chaperones and is involved in a variety of protein homeostasis regulation. It was identified to prevent intracellular aggregation of toxic polyglutamine peptide [66] and also inhibit the aggregation of another AD pathognomonic protein, Aβ42 [67]. Furthermore, a conserved S/T-rich region in the C-terminal domain of DNAJB6 is critical for suppressing the aggregation of Huntingtin oligomers [38]. Our DNAJB6-tau findings extend the current knowledge about the clients of DNAJB6. In general, JDPs are highly specialized with very little or no functional redundancy with other J-domain proteins [68, 69]. Based on this, we surmise that DNAJB6 might be the major JDP to assist the delivery of tau. The results reinforce the pivotal role of DNAJB6 in assisting in the folding of divergent client proteins related to neurodegenerative diseases.

Numerous neurodegenerative diseases are not caused by genetic mutations [70]. And genetic mutations only contribute to less than 5% of the cause of ADs. Many pieces of evidence suggest that environmental factors in daily life may play a major contributor. Head trauma, air pollution, poor sleep, and excessive alcohol consumption can all be a source of stress. Stress may lead to a decline in JDP expression. Indeed, in a large-scale gene expression study, it was demonstrated that hypoxia can trigger a decrease in DNAJB6 expression [71–73]. Brain damage often causes ischemia and hypoxia [74], which may therefore increase the risk of the formation of ADs.

Many stresses come from the external environment. Of course, this also indirectly causes changes in the internal signal transduction, which may lead to alteration of cellular epigenetic regulation and gene expression. miR-632 was found to be a potentially important epigenetic regulator of DNAJB6, which contributes to the downregulation of DNAJB6 [75]. Sometimes stresses can change many enzymes’ activities, such as kinase alteration and/or posttranslational modification (PTM) of proteins. The PTMs of DNAJB6 are well described in the PhosphoSitePlus website, which summarizes phosphorylation, ubiquitylation, and acetylation sites on DNAJB6 from high-throughput data sources. Although currently, there are no detailed functional analyses on DNAJB6’s posttranslational modifications, we believe that DNAJB6b is likely to be posttranslationally modified, resulting in changes in its ability to deliver its clients and/or to trigger HSP70’s function. Considering our findings, altering DNAJB6b expression and regulations might be a possible strategy to prevent AD. These directions should be worthy of examination and angles to be explored in future research and validated in post-mortem brain tissues from patients with AD.

## Conclusions

Overall, this study identifies that DNAJB6b recognizes tau and helps to deliver it to the cytoplasmic HSP70 folding system. Inverse correlations between DNAJB6 and pathological phospho-tau expression in an age-dependent manner in a mouse model of tau pathology further strengthen our findings that DNAJB6b might be a direct sensor to avoid tau aggregation in neuronal cells, thus helping to prevent AD.

## Methods

### Cell lines

HEK-293 T cells were obtained from the American Type Culture Collection (ATCC) and maintained in Dulbecco’s modified Eagle’s medium (DMEM) high glucose (Cytiva, Logan, UT, USA) supplied with 10% fetal bovine serum, 1 × penicillin/streptomycin/fungizone, 1 mM sodium pyruvate, and 1 × nonessential amino acids. SH-SY5Y cells were obtained from ATCC and cultured in DMEM/F12 medium (Cytiva) supplied with 10% fetal bovine serum and 1 × penicillin/streptomycin/fungizone. All cell lines were cultured under standard conditions (37 °C, 5% CO_2_) and routinely tested for the absence of mycoplasma. For inducing aggregation of α-synuclein in SH-SY5Y cells, cells were treated with 100 nM rotenone (Sigma-Aldrich, Louis, MO, USA) for 24 h before analysis.

### Transfection

For transient overexpression, transfection was conducted using T-Pro non-liposome transfection reagent II (T-Pro Biotechnology, New Taipei City, Taiwan) for 293 T cells and Lipofectamine™ LTX reagent with PLUS™ reagent (Thermo Fisher Scientific, Waltham, MA, USA) for SH-SY5Y cells following the manufacturer instructions.

### Lentivirus packaging and infection

HEK-293 T cells were co-transfected with the packaging plasmid (pCMV-Δ8.91), envelope (pMD.G) and either hairpin pLKO-RNAi vectors (National RNAi Core Facility, Institute of Molecular Biology/Genomic Research Centre, Academia Sinica, Taiwan) for the virus production. The specific oligonucleotide sequences of shRNA are listed in Additional file 1: Table S1. After 24 h post-transfection, the medium was replaced by DMEM containing 1% BSA medium. Virus-containing supernatants were collected after 48 h and 72 h post-transfection. For a 6-well culture, 3 × 10^5^ SH-SY5Y cells were infected with each virus plus DMEM/F12 medium containing 1 μg/ml polybrene (Millipore, Billerica, MA, USA) for 16 h. The transduced cells were selected with DMEM/F12 medium containing 1 μg/ml puromycin (Sigma-Aldrich) for the indicated days.

### Plasmids

Primers used for plasmid generation are listed in Additional file 1: Table S2. For the pEGFP-C1-tau (2N4R) construction, *tau* cDNA (2N4R) was amplified from the expression plasmid containing a Myc-tagged human four-repeat tau [36] (a gift from Dr. Akihiko Takashima) by PCR using primers tau-XhoI-For and tau-KpnI-Rev and then cloned into the XhoI-KpnI sites of pEGFP-C1, following the N-terminal EGFP-tag. The VN-tau (P301L) (Addgene, Watertown, MA, USA), tau (P301L)-VC (Addgene), pcDNA5/FRT/TO-V5-DNAJB6a (Addgene), and pcDNA/FRT/TO-V5-DNAJB6b (Addgene) were purchased from Addgene. The pEGFP-C1-tau P301L (2N4R) and pcDNA5/FRT/TO-V5-DNAJB6b H31Q plasmids were generated by site-directed mutagenesis using HiFi polymerase (Kapa Biosystems, Wilmington, MA, USA) with primers, tau-P301L-AvrII-For and DNAJB6-H31Q-AflII-For, respectively. All plasmids were sequenced before being used and are listed in Additional file 1: Table S3.

### Filter trap assay

The Triton-fractionation assay was performed as previously described [33, 76]. Cells were harvested and lysed with filter trap lysis buffer (1% Triton X-100 in 1 × PBS, pH 7.4) containing 1 mM PMSF and Complete EDTA-free Protease Inhibitor Cocktail (Roche, Basel, Switzerland), followed by brief sonication. The protein concentration was determined by the Bio-Rad Protein Assay (Bio-Rad Laboratories, Hercules, CA, USA). Before filtering, the samples were diluted to a final concentration of 1 μg/μl with filter trap lysis buffer containing 1% sodium dodecyl sulfate (SDS). The samples were then filtered through 0.2-μm cellulose acetate membranes (Sterlitech, Auburn, WA, USA), using a 96-well dot-blot apparatus (Bio-Rad Laboratories). Before filtration, the membranes were immersed in rinse buffer (1% SDS in 1 × PBS, pH 7.4). Filter dots were washed once with 0.05% TBST (150 mM NaCl, and 0.05% Tween 20, 20 mM Tris–HCl, pH 7.4). Proteins trapped by the filter were detected by immunostaining following the procedure of immunoblotting described below.

### Co-immunoprecipitation assay

Cells were harvested and lysed in immunoprecipitation (IP) buffer (100 mM NaCl, 0.5% Triton X-100, 1 mM EDTA, 1 mM PMSF, 50 mM Tris–HCl, pH 7.5), supplemented with Complete EDTA-free Protease Inhibitor Cocktail (Roche). Anti-DNAJB6 antibody (Abcam, Cambridge, UK) or anti-V5 antibody (Thermo Fisher Scientific) was added to a final concentration of 2 μg/ml lysate and incubated overnight at 4 °C. Lysates were then incubated with protein G Mag Sepharose Xtra magnetic beads (Cytiva) for 3 h at 4 °C. After extensive washing with IP buffer three times, the bound proteins were eluted with 60 μl of 2 × SDS sample buffer (250 mM Tris, pH 6.8, 10% SDS, 0.25% bromophenol blue, 50% sucrose, 0.5 M 2-mercaptoethanol). Precipitates were then analyzed by immunoblotting with appropriate antibodies.

### Subcellular fractionation

The Triton-fractionation assay was performed as previously described [34, 35]. Cells were harvested, washed once with 1 × PBS, and lysed with 400 μl Triton Lysis buffer (25 mM Tris–HCl, pH 7.5, 150 mM NaCl, 1 mM EDTA, 1% Triton X-100, 20 mM NaF, and 1 mM PMSF) containing Complete EDTA-free Protease Inhibitor Cocktail (Roche); 500 μg of cell lysates were centrifuged at 20,000 × *g* for 30 min at 4 °C, and the supernatants were collected. To ensure the complete removal of the supernatant, the pellets were washed with 400 μl Triton Lysis buffer and underwent 20,000 × *g* centrifugation. After that, the supernatants were completely removed, and the pellets were resuspended in a 200 μl Triton Lysis buffer; 5 × SDS sample buffer was then added to both the Triton Lysis buffer-soluble and buffer-insoluble fractions, and the samples were heated at 100 °C for 10 min. The Triton-insoluble fraction was probe-sonicated and boiled again for 10 min to ensure homogeneity.

### Immunoblotting

Whole proteins were extracted and resolved by SDS–polyacrylamide gel electrophoresis (SDS-PAGE) and transferred to a polyvinylidene difluoride membrane (PVDF) (Millipore). The membrane was blocked in 5% nonfat milk at room temperature for 1 h, followed by incubation with primary antibodies at 4 °C overnight. Primary antibodies were used to detect tau (1:1000, GeneTex, Irvine, CA, USA), HSPA8 (1:1000, Novus Biologicals, Centennial, CO, USA), HSPA1A (1:1000, Santa Cruz, Dallas, TX, USA), BiP (1:1000, Abcam), HSPA9 (1:1000, Thermo Fisher Scientific), DNAJB6 (1:1000, Abcam), β-actin (1:1000, Proteintech, Rosemont, IL, USA), V5 (1:1000, Thermo Fisher Scientific), α-synuclein (1:1000, GeneTex), and DNAJA3 (1:500, Santa Cruz, Dallas, TX, USA). Signals were developed using Luminata™ Crescendo Western HRP Substrate (Millipore). The image was quantified by ImageJ software.

### Bimolecular fluorescence complementation assay

The BiFC assay was performed according to a previous study [41]. SH-SY5Y cells were co-transfected for 48 h with plasmids that encode tau P301L protein fused to the N-terminal part of Venus protein, VN-tau P301L (Addgene), and tau P301L protein fused to the C-terminal part of Venus protein, tau P301L-VC (Addgene). The transfection efficiency of these two plasmids was verified by immunoblotting with an anti-tau antibody (GeneTex,). For obtaining representative images, the fluorescence of reconstituted Venus protein was analyzed using a Zeiss ApoTome.2 microscope. For quantifying the percentage of cells developing tau aggregates, the images were taken using a Zeiss Imager.M2 fluorescence microscope.

### Proximity ligation assay

To determine the in situ interaction of DNAJB6b and tau, the PLA [54, 55] was performed according to the manufacturer’s protocol with some modifications (Merck, Darmstadt, Germany). SH-SY5Y cells grown on slide coverslips (⏀15 mm) were washed with pre-cooled PBS and then fixed with 4% paraformaldehyde for 10 min at room temperature, followed by cell permeabilization with PBS containing 0.1% Triton-X-100 for 10 min at room temperature. The cells on the coverslips were faced down and immersed in the blocking solution (DUO82007, Merck) for 1 h at 37℃ in a humidity incubator. After blocking, cells were incubated with primary antibodies (anti-V5, 1:250, R960-25, Thermo Fisher Scientific, and anti-tau, 1:250, GTX112981, Genetex) overnight at 4℃. On the next day, the cells were washed twice with Duolink Wash Buffer A (DUO82049, Merck) and incubated with Duolink PLA probes (anti-rabbit minus, DUO92005, and anti-mouse plus, DUO92001, Merck) for 1 h at 37℃. The subsequent ligation and amplification were carried out using Detection Reagents Red (DUO92008, Merck). The cells were then washed twice with Duolink Wash Buffer B (DUO82049, Merck), and the nuclear DNA was stained by 4', 6-diamidino-2-phenylindole (DAPI) (Thermo Fisher Scientifics). The coverslips were mounted with Fluoromount™ Aqueous Mounting Medium (Sigma-Aldrich) overnight at room temperature.

### Animal study

The rTg4510 transgenic mice expressing four-repeat human tau with the P301L mutation [57] were kindly provided by APRINOIA Therapeutics (APRINOIA Therapeutics Inc., Taipei, Taiwan) licensed from the Mayo Clinic and bred at the National Laboratory Animal Center (NLAC, Taipei Taiwan). Animals were placed on a standard rodent diet ad libitum and housed under a 12-h/12-h light–dark cycle. The animal study was approved by the Institutional Animal Care and Use Committee at the Laboratory Animal Center of NLAC and the College of Medicine of National Taiwan University.

### Immunohistochemistry staining

The heterozygous rTg4510 male mice and the littermate wild-type control male mice were sacrificed at the age of 3 and 6.5 months old individually. Dissected mouse brains were fixed in 4% buffered formaldehyde, followed by dehydration, and then embedded in paraffin and sliced into 5-μm sections. The sections were de-waxed and labeled with the following antibodies: DNAJB6 (1:200, ab198995, Abcam) and phospho-tau at Ser 202 and Thr 205 residues (AT8, 1:100, MN1020, Thermo Fisher Scientifics) for 24 h at 4 °C. After rinsing in 0.1 M sodium phosphate buffer, slices were incubated for 2 h at room temperature in solutions containing a goat anti-rabbit antibody coupled to Alexa Fluor 647 (1:500, Thermo Fisher Scientifics) and a goat anti-mouse coupled to Alexa Fluor 488 (1:500, Thermo Fisher Scientifics). Sections were examined with a Zeiss LSM 710 confocal laser scanning system (Carl Zeiss Microscopy GmbH, Jena, Germany). Fluorescence emission was recorded through separated channels. The individual CA1 and CA3 regions were analyzed using the composite images combining the confocal image stacks recorded through the different channels using Zen software (ZEN 2012, Carl Zeiss Microscopy). Two 200 mm × 200 mm regions centered at the hippocampal CA1 or CA3 stratum pyramidale layer were analyzed and averaged in each section. Immunofluorescence intensity of DNAJB6 or AT8 was quantified and the background signal was subtracted by Image J (National Institutes of Health, Bethesda, MD). The results were normalized to the mean of the 3 M littermate wild-type control group.

### RNA purification and quantitative reverse transcription PCR

RNA was extracted using TRIzol reagent (Thermo Fisher Scientifics). cDNA was synthesized using Thermo Scientific Maxima First Strand cDNA Synthesis (Thermo Fisher Scientific). qRT-PCR was performed on a BioRad CFX Connect Real-Time PCR Detection System. All primer sequences for PCR are listed in Additional file 1: Table S4.

### Other information

The images of the full immunoblots are in Additional file 1: Figure S10. The other detailed information on materials used in this study is listed in tables of Additional file 1. Antibodies: Additional file 1: Table S5; Chemicals and Reagents: Additional file 1: Table S6; Critical commercial assays: Additional file 1: Table S7; Software: Additional file 1: Table S8; Other materials and instruments: Additional file 1: Table S9.

### Quantification and statistical analysis

The protein levels in immunoblotting and dot blot assays were quantified using Image J and at least three independent experiments were performed. For quantifying the percentage of cells developing tau aggregates indicated by tau BiFC assay, 10–15 random-field images were taken at × 10 magnification on a Zeiss Imager.M2 fluorescence microscope for each condition, and three independent experiments were performed. For quantifying the percentage of cells developing PLA positive signal, 5 random-field images were taken at × 10 magnification on a Zeiss Imager.M2 fluorescence microscope for each condition, and three independent experiments were performed.

All experiments were performed in a minimum of three biological replicates. The applied statistical tests as well as the number of replicates (*n*) are specified in the relevant figure legends. *F*-tests were conducted to test for significant differences in the variance of each sample (significant at *P* < 0.05). When variances were not different, unpaired two-tailed Student’s *t*-tests were performed, and when variances were different, unpaired two-tailed Welch’s *t*-tests were performed. All of the continuous variables are expressed as the mean ± S.D. *P*-values < 0.05 were considered statistically significant and were indicated in the figures. (*, *P* < 0.05, **, *P* < 0.01, ***, *P* < 0.001, ****, *P* < 0.0001).

### Supplementary Information

**Additional file 1: Figures S1-S10 **and** Tables S1-S9. Figure S1. **P301L mutation of tau increases the insoluble form of tau in the cells.** Figure S2. **Knockdown efficiency of JDPs in SH-SY5Y cells.** Figure S3. **DNAJB6-knockdown cells display increased tau aggregation.** Figure S4. **Knockdown of DNAJB6 does not increase α**-**synuclein aggregation in SH-SY5Y cells**. Figure S5. **DNAJB6 reduces the insoluble form of tau in the cells. **Figure S6. **Knockdown of DNAJB6 does not alter the protein levels of other molecular chaperones.** Figure S7. **Overexpression of tau P301L mutant and knockdown of DNAJB6 induce caspase-9-dependent apoptosis pathway.** Figure S8. **Overexpression of DNAJB6 does not alter the protein levels of other molecular chaperones.** Figure S9. **DNAJB6b is critical for preventing tau aggregation.** Figure S10. **Images of the full immunoblots.** Table S1. **Oligonucleotide sequences of shRNA.** Table S2. **Primers used for plasmid generation.** Table S3. **Plasmids.** Table S4. **Primer sequences for PCR. Table S5. Antibodies. **Table S6.** Chemicals and Reagents.** Table S7. **Critical commercial assays.** Table S8. **Software.** Table S9. **Other materials and instruments.

**Additional file 2. **The individual data values for Figure. 1B, E, 2D, 3C, 4C, 5C, E, 6B, E-F, 7E, S1B, S2, S3B, S4B, S5D-F, S6B-E, S7B, S8B-E.

## Data Availability

All data generated or analyzed during this study are included in this published article and its supplementary information files. The individual data values of the replicates in the article and Additional file 1 are listed in Additional file 2. All materials generated in this study are available from the corresponding authors with a completed Materials Transfer Agreement.

## Abbreviations

AD: Alzheimer’s disease
BiFC: Bimolecular fluorescence complementation
NLS: Nuclear localization signal
PLA: Proximity ligation assay
HSP: Heat shock protein
JDPs: J-domain proteins
PTM: Posttranslational modification
shLuc: Knockdown of firefly luciferase
HPD: Histidine-proline-aspartate
DMEM: Dulbecco’s modified Eagle’s medium
ATCC: American Type Culture Collection
IP: Immunoprecipitation
SDS-PAGE: SDS-polyacrylamide gel electrophoresis
PVDF: Polyvinylidene difluoride membrane
DAPI: 4', 6-Diamidino-2-phenylindole
qRT-PCR: Quantitative reverse transcription PCR
