# Supplementary material for: The HSP40 family chaperone isoform DNAJB6b prevents neuronal cells from tau aggregation
Source: BMC Biol. 2023 Dec 18;21:293. doi: 10.1186/s12915-023-01798-6 (PMC10729500; doi:10.1186/s12915-023-01798-6)
Supplement: Supplementary file 1 — Additional file 1: Figures S1-S10 and Tables S1-S9. Figure S1. P301L mutation of tau increases the insoluble form of tau in the cells. Figure S2. Knockdown efficiency of JDPs in SH-SY5Y cells. Figure S3. DNAJB6-knockdown cells display increased tau aggregation. Figure S4. Knockdown of DNAJB6 does not increase α-synuclein aggregation in SH-SY5Y cells. Figure S5. DNAJB6 reduces the insoluble form of tau in the cells. Figure S6. Knockdown of DNAJB6 does not alter the protein levels of other molecular chaperones. Figure S7. Overexpression of tau P301L mutant and knockdown of DNAJB6 induce caspase-9-dependent apoptosis pathway. Figure S8. Overexpression of DNAJB6 does not alter the protein levels of other molecular chaperones. Figure S9. DNAJB6b is critical for preventing tau aggregation. Figure S10. Images of the full immunoblots. Table S1. Oligonucleotide sequences of shRNA. Table S2. Primers used for plasmid generation. Table S3. Plasmids. Table S4. Primer sequences for PCR. Table S5. Antibodies. Table S6. Chemicals and Reagents. Table S7. Critical commercial assays. Table S8. Software. Table S9. Other materials and instruments. [file 12915_2023_1798_MOESM1_ESM.docx]

**Additional file 1**

**
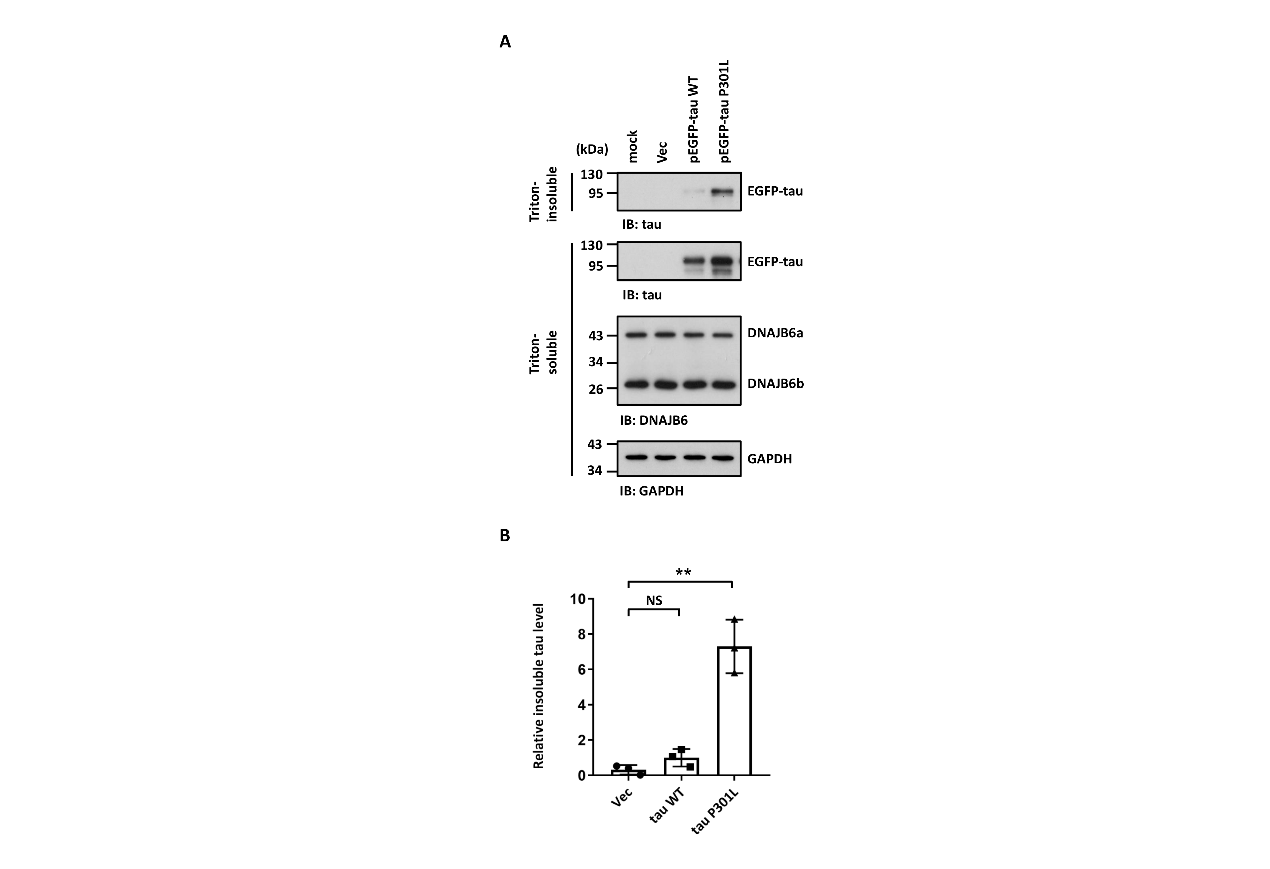
**

**Figure S1. P301L mutation of tau increases the insoluble form of tau in the cells.**

**(A)** SH-SY5Y neuroblastoma cells were transfected with the empty vector, wild-type tau, or mutated tau P301L. Cells were harvested at 48 hrs post-transfection. The cell lysates were biochemically fractionated into Triton-soluble and -insoluble fractions as described in “Methods”. The Triton-soluble and -insoluble fractions were analyzed by immunoblotting with a total tau antibody. DNAJB6 expression levels were analyzed by immunoblotting with a DNAJB6 antibody. The GAPDH was used as a loading control. **(B)** Quantification of the Triton-insoluble form of tau shown in (A). The Triton-insoluble form of tau was normalized to GAPDH in the respective Triton-soluble fraction. The values were given as mean ± S.D. (n=3, *P< 0.05, **P< 0.01, unpaired two-tailed Student’s t-test. The individual data values of the replicates in (B) are listed in Additional file 2.

**
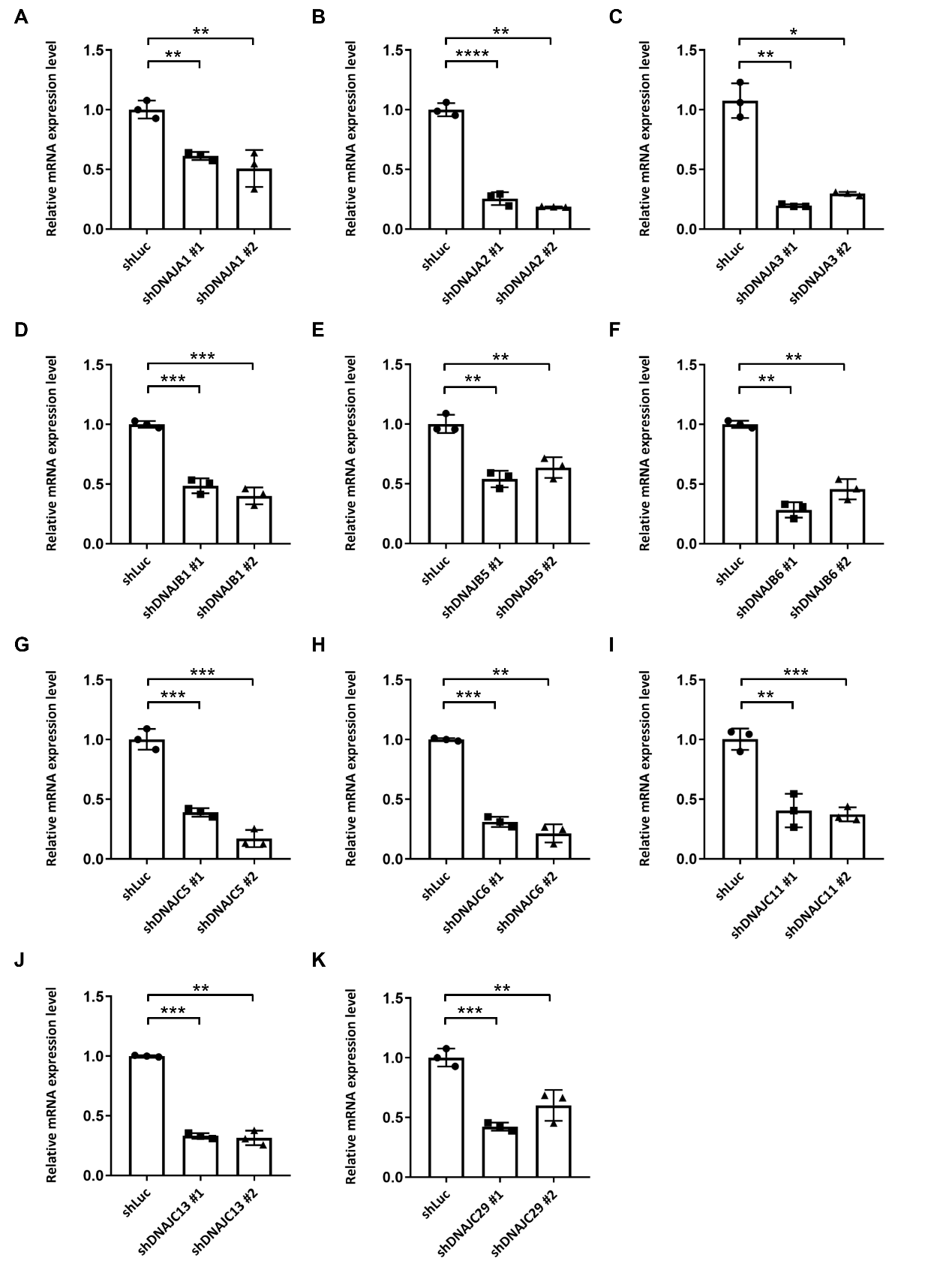
**

**Figure S2. Knockdown efficiency of JDPs in SH-SY5Y cells.**

**(A-K)** The endogenous mRNA levels of eleven JDPs in shLuc and shJDPs knockdown cells were analyzed by qRT-PCR and normalized to respective mRNA levels of ribosomal protein L (RPL). The values were given as mean ± S.D. (n=3, *P< 0.05, **P< 0.01, ***P< 0.001, ****P< 0.0001, unpaired two-tailed Student’s t-test (A, D-G, I, K), unpaired two-tailed Welch’s t-test (B-C, H, J)). The individual data values of the replicates in (A-K) are listed in Additional file 2.


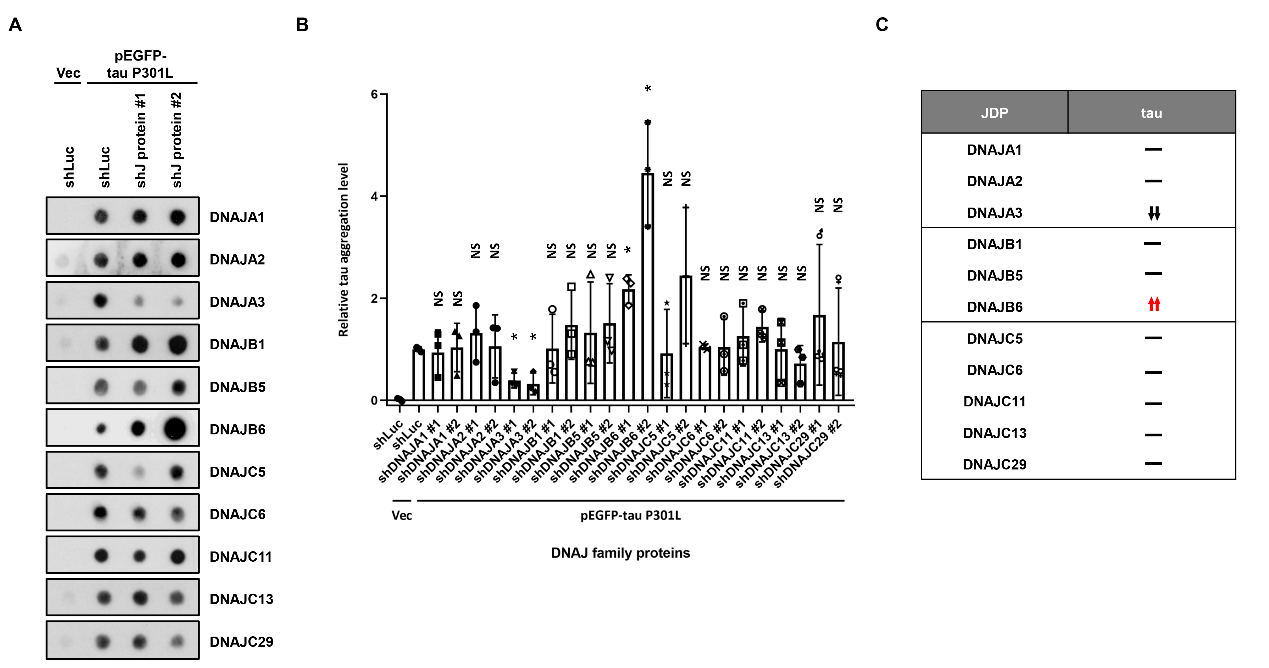


**Figure S3. DNAJB6-knockdown cells display increased tau aggregation.**

**(A)** shLuc or shJDPs SH-SY5Y cells were transfected with the empty vector or tau P301L for 48 hrs. The cellular lysate of SH-SY5Y cells was filtered through cellulose acetate membranes and retained proteins were stained with a tau antibody. Two shRNA clones were analyzed for each JDP. Three biological replicates were performed for each shRNA, and the images presented here are representatives of the replicates. **(B)** Quantification of aggregated tau. The tau aggregation levels in JDP-knockdown cells were compared with those in shLuc cells. The values were given as mean ± S.D. (n=3, *P< 0.05, unpaired two-tailed Welch’s t-test). The individual data values of the replicates in (B) are listed in Additional file 2. **(C)** The screening summary of tau aggregation of the eleven neurodegenerative disease-related JDPs.


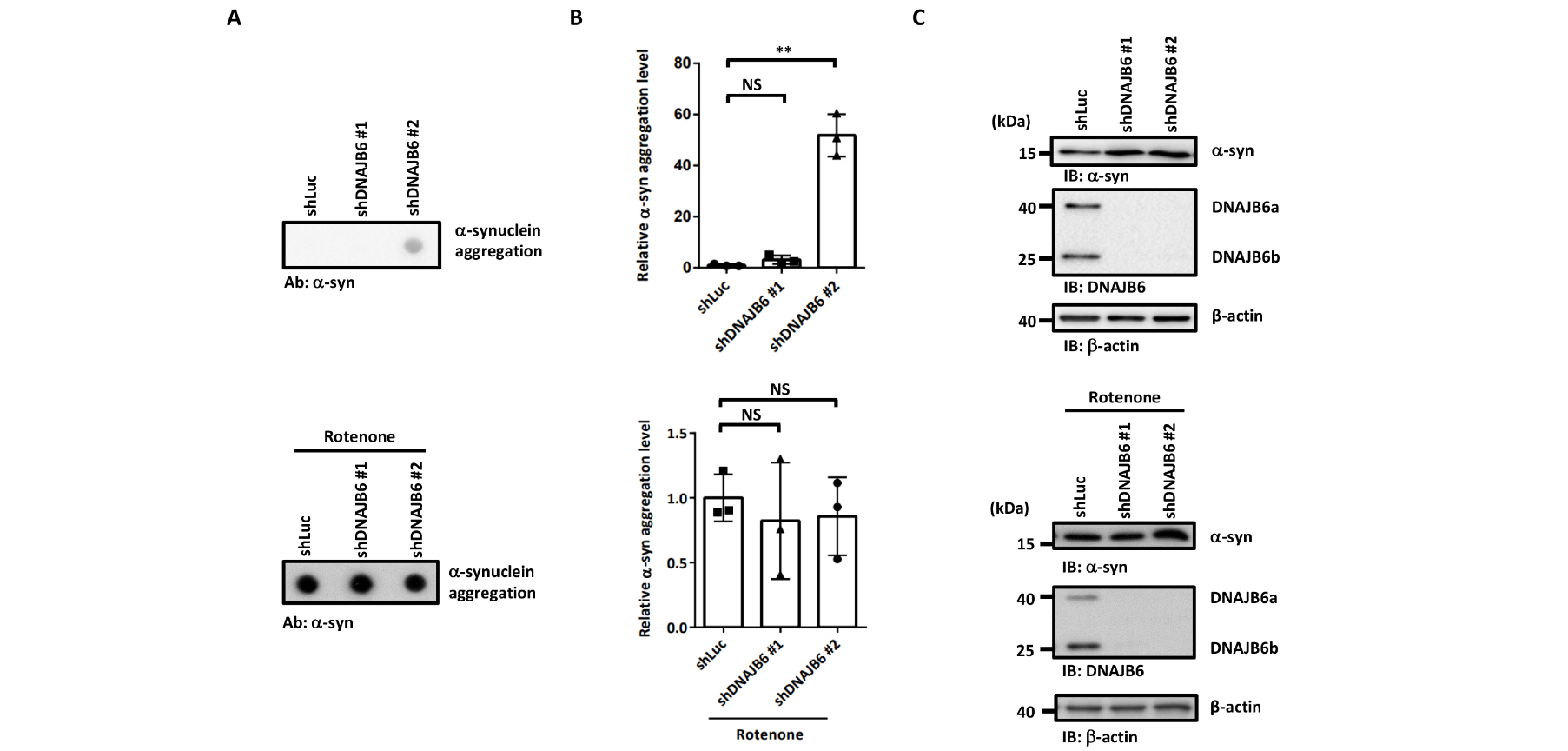


**Figure S4. Knockdown of DNAJB6 does not increase α-synuclein aggregation in SH-SY5Y cells.**

**(A)** shLuc or shDNAJB6 SH-SY5Y cells were treated with solvent or 100 nM rotenone for 24 hrs. The cellular lysate was filtered through a nitrocellulose membrane and retained proteins were stained with an α-synuclein antibody. (B) Quantification of aggregated α-synuclein. β-actin shown in (C) serves as a loading control. The values were given as mean ± S.D. (n=3, unpaired two-tailed Student’s t-test). **(C)** The expression levels of α-synuclein and DNAJB6 were determined by immunoblotting. The individual data values of the replicates in (B) are listed in Additional file 2.

**
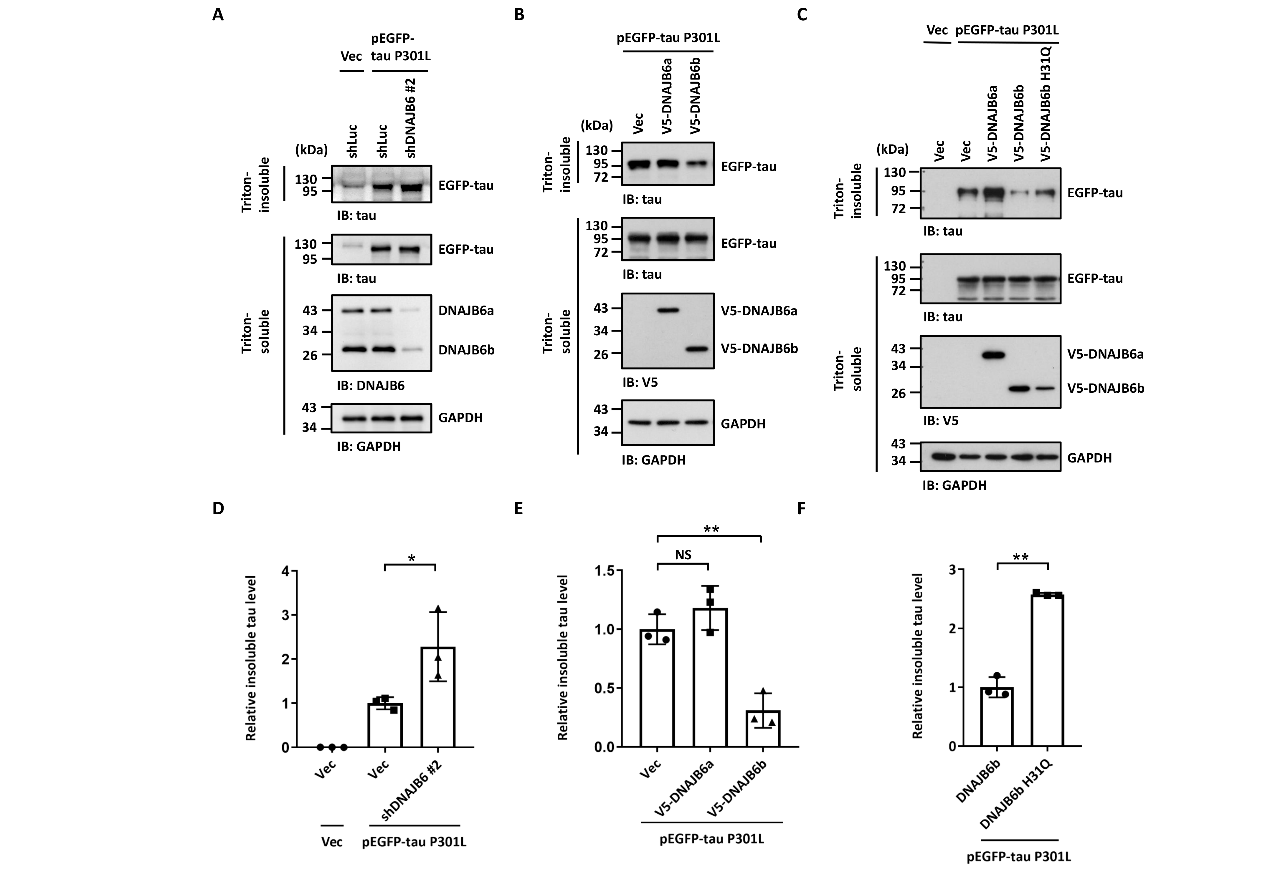
**

**Figure S5. DNAJB6 reduces the insoluble form of tau in the cells.**

**(A)** shLuc or shDNAJB6 SH-SY5Y neuroblastoma cells were transfected with the empty vector or tau P301L for 48 hrs. The cell lysates were biochemically fractionated into Triton-soluble and -insoluble fractions. The Triton-soluble and -insoluble fractions were analyzed by immunoblotting with a total tau antibody. DNAJB6 expression levels were analyzed by immunoblotting with a DNAJB6 antibody. The GAPDH was used as a loading control. **(B)** SH-SY5Y cells were co-transfected with the empty vector or mutated tau P301L together with the empty vector, V5-DNAJB6a, or V5-DNAJB6b for 48 hrs. **(C)** SH-SY5Y cells were co-transfected with the mutated tau P301L together with the empty vector, V5-DNAJB6b or V5-DNAJB6b H31Q mutation for 48 hrs. **(D-F)** Quantification of the Triton-insoluble form of tau shown in (A-C), respectively. The Triton-insoluble form of tau was normalized to GAPDH in the respective Triton-soluble fraction. The values in (D-F) were given as mean ± S.D. (n=3, *P< 0.05, **P< 0.01, unpaired two-tailed Student’s t-test (D-E), unpaired two-tailed Welch’s t-test (F)). The individual data values of the replicates in (D-F) are listed in Additional file 2.


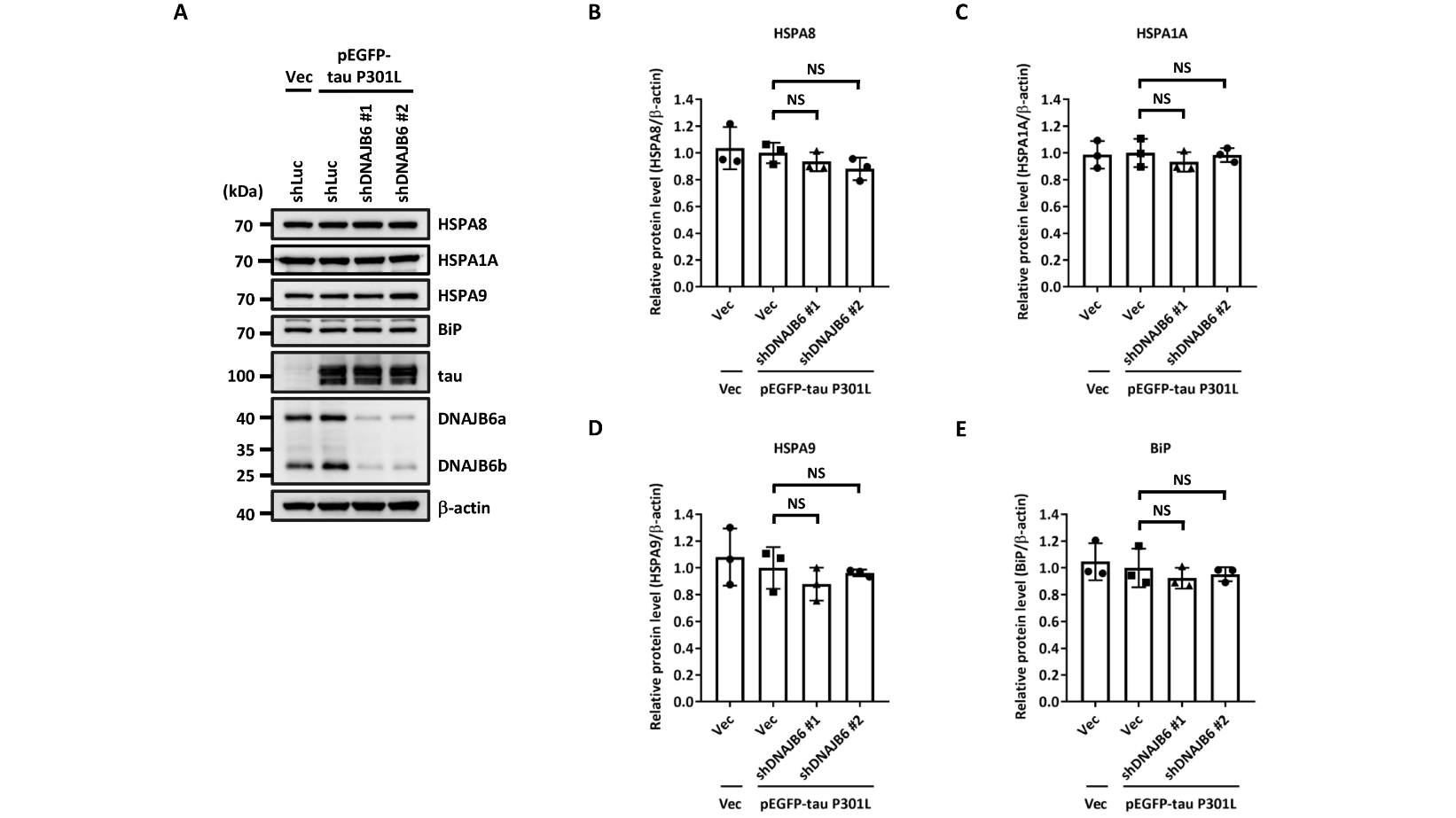


**Figure S6. Knockdown of DNAJB6 does not alter the protein levels of other molecular chaperones.**

**(A)** shLuc or shDNAJB6 SH-SY5Y cells were transfected with the empty vector or tau P301L for 48 hrs. Different chaperones, HSPA8, HSPA1A, HSPA9, and BiP were detected by immunoblotting. β-actin was used as a loading control. **(B-E)** Quantification of the protein levels of HSPA8 (B), HSPA1A (C), HSPA9 (D), and BiP (E) showed in (A) were normalized to internal control, β-actin. The values were given as mean ± S.D. (n=3, unpaired two-tailed Student’s t-test). The individual data values of the replicates in (B-E) are listed in Additional file 2.


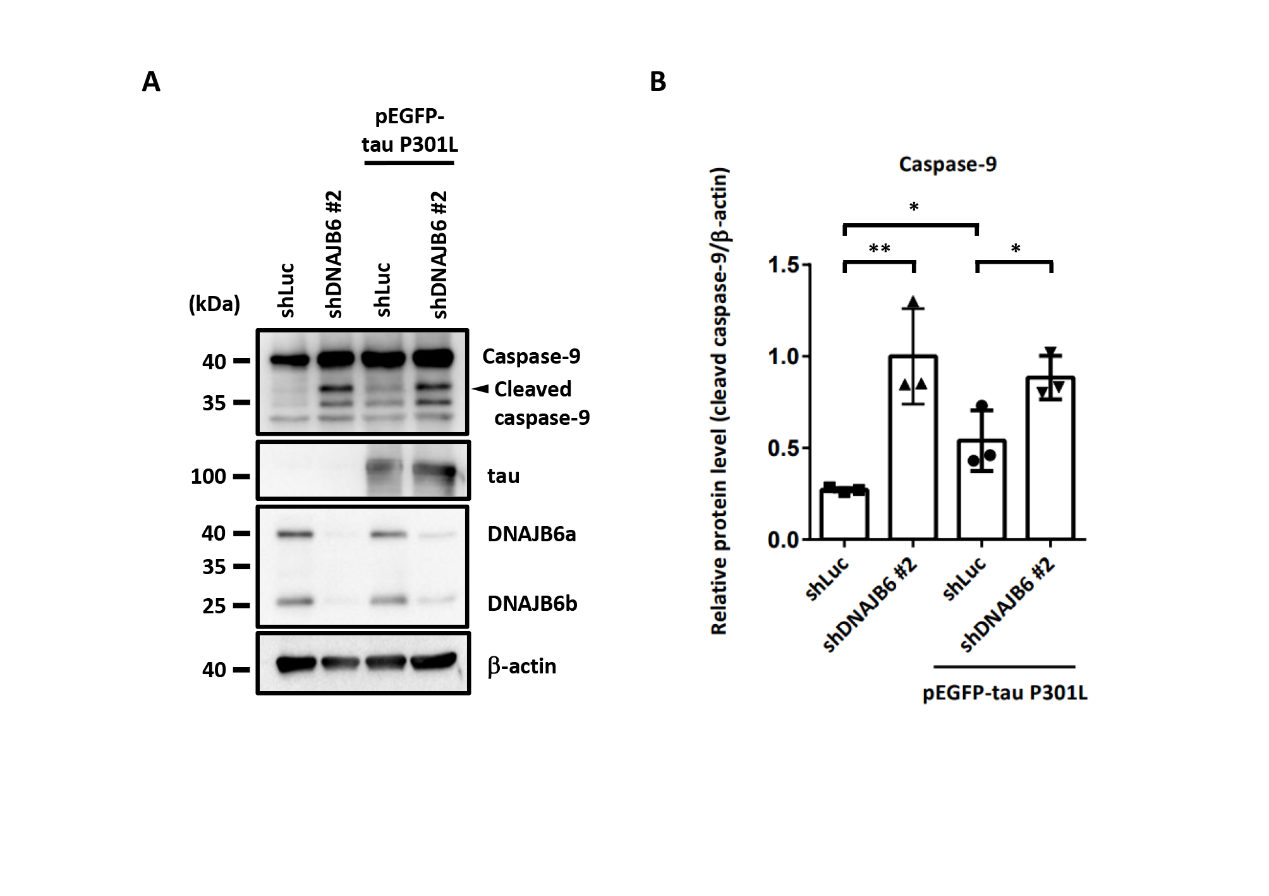


**Figure S7. Overexpression of tau P301L mutant and knockdown of DNAJB6 induce caspase-9-dependent apoptosis pathway.**

**(A)** shLuc or shDNAJB6 SH-SY5Y cells were transfected with the empty vector or tau P301L mutant for 48 hrs. The cellular apoptotic marker, cleaved caspase-9, was detected by immunoblotting. β-actin was used as a loading control. **(B)** The protein levels of 37 kDa cleaved caspase-9 were quantified and normalized to the internal control. The values were given as mean ± S.D. (n=3, *P< 0.05, unpaired two-tailed Student’s t-test). The individual data values of the replicates in (B) are listed in Additional file 2.


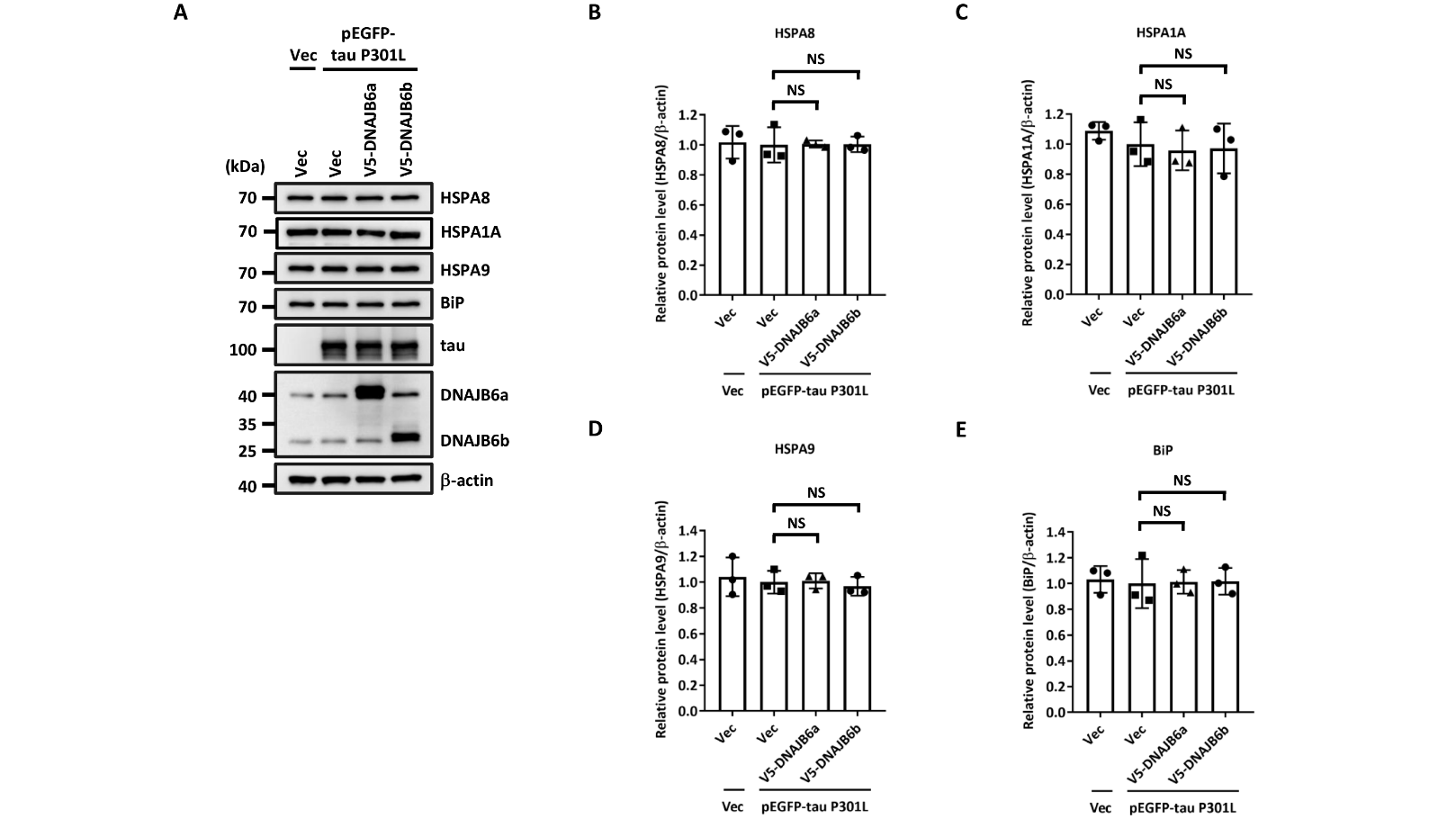


**Figure S8. Overexpression of DNAJB6 does not alter the protein levels of other molecular chaperones.**

**(A)** SH-SY5Y cells were co-transfected with an empty vector or mutated tau P301L together with an empty vector (pcDNA/FRT/TO-V5), V5-DNAJB6a, or V5-DNAJB6b for 48 hrs. Different chaperones, HSPA8, HSPA1A, HSPA9, and BiP were detected by immunoblotting. β-actin was used as a loading control. **(B-E)** The protein levels of HSPA8 (B), HSPA1A (C), HSPA9 (D), and BiP (E) as shown in (A) were quantified and normalized to the internal control, β-actin. The values were given as mean ± S.D. (n=3, unpaired two-tailed Student’s t-test). The individual data values of the replicates in (B-E) are listed in Additional file 2.

**
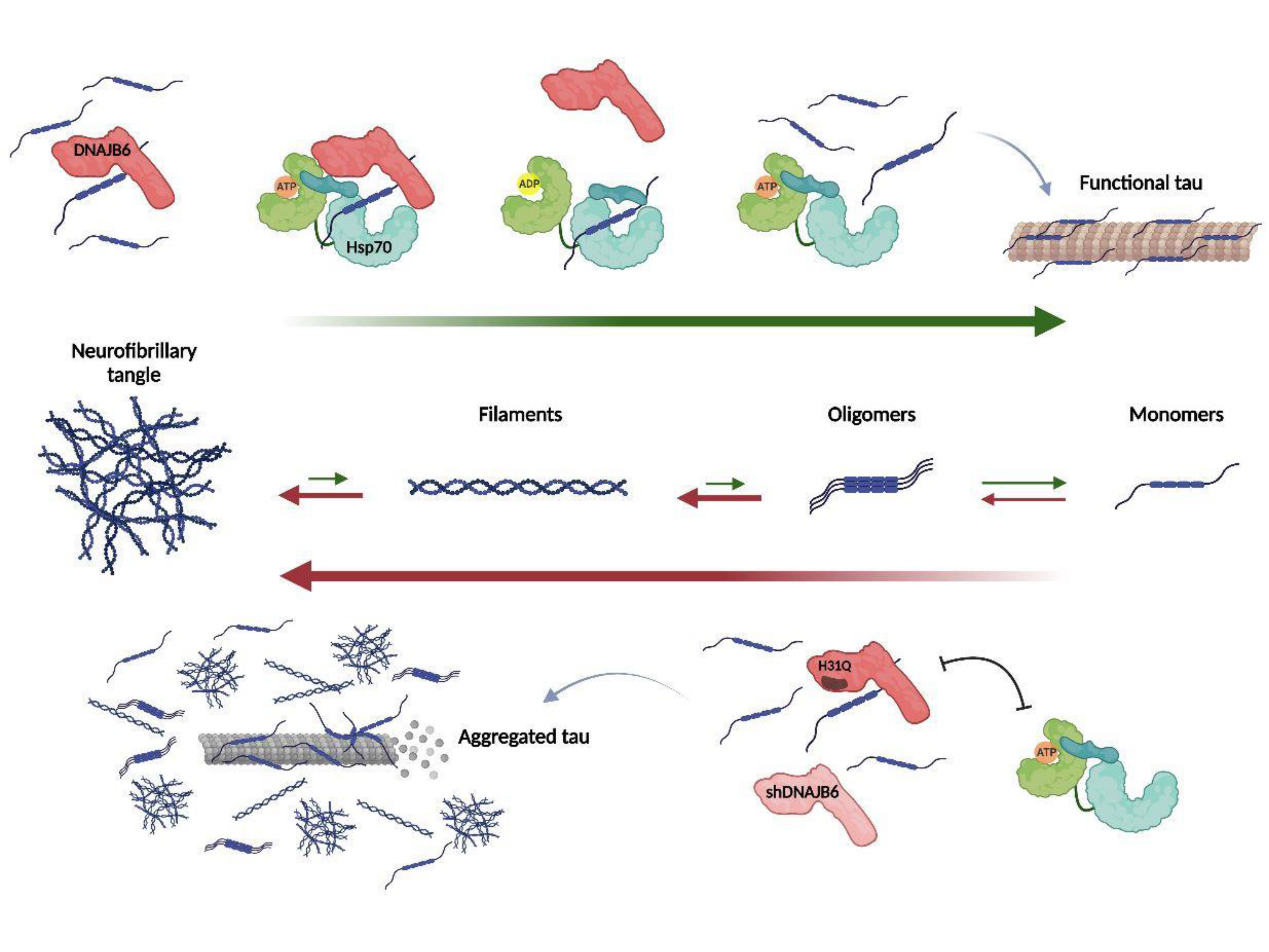
**

**Figure S9. DNAJB6b is critical for preventing tau aggregation.**

DNAJB6b delivers tau to the HSP70 folding system in the neuronal cell cytoplasm. Hydrolysis of ATP triggers the conformation change to close the lid of HSP70. The nucleotide exchange factor replaces ADP with ATP to release the cargo tau for microtubulin assembly. Knockdown of DNAJB6b may accumulate insoluble cytoplasmic tau oligomers, which then tend to aggregate and ultimately lead to the formation of neurofibrillary tangles. Years of this accumulation of aggregates may destroy the function of the cytoskeleton of neuronal cells in the brain and strengthen AD.

**
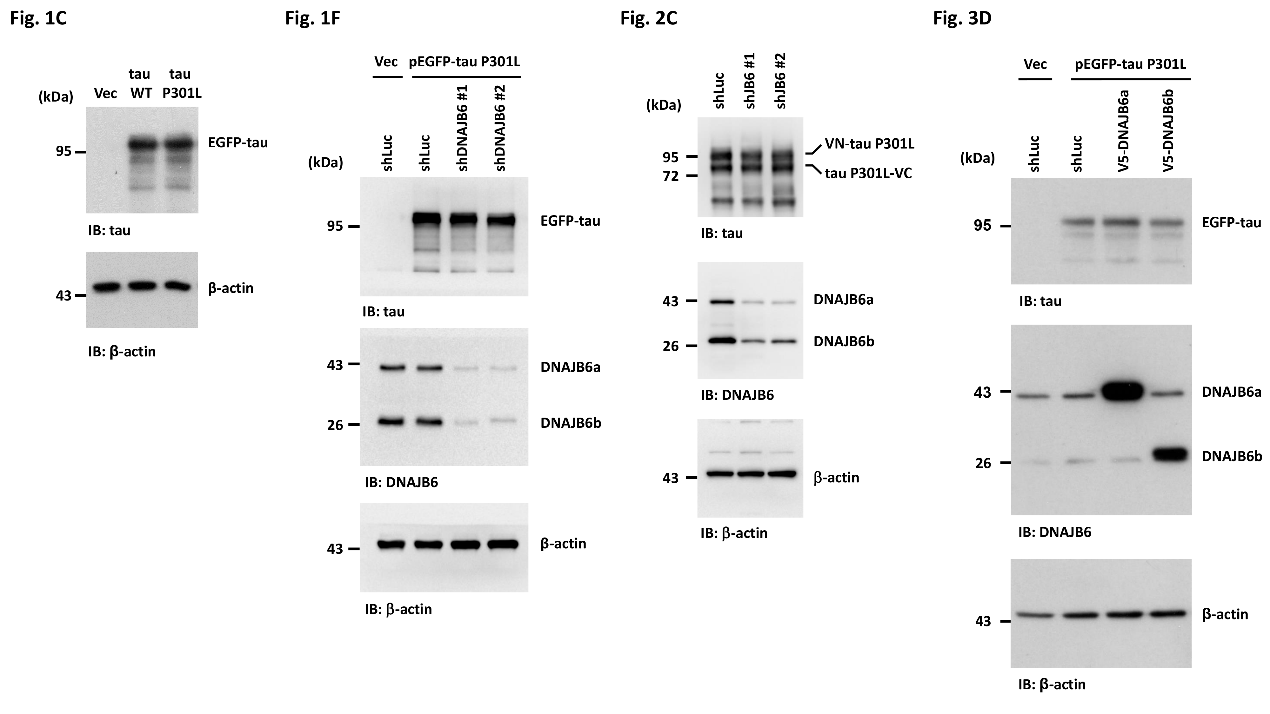
**

**
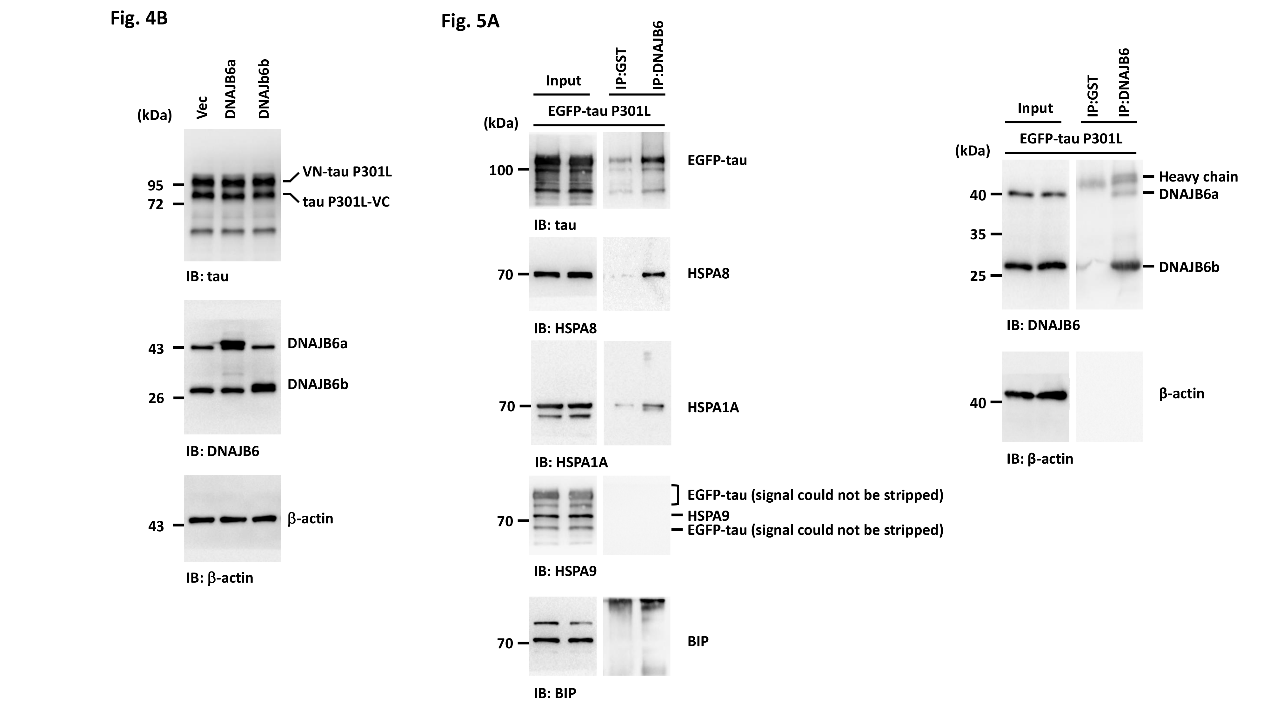
**

**
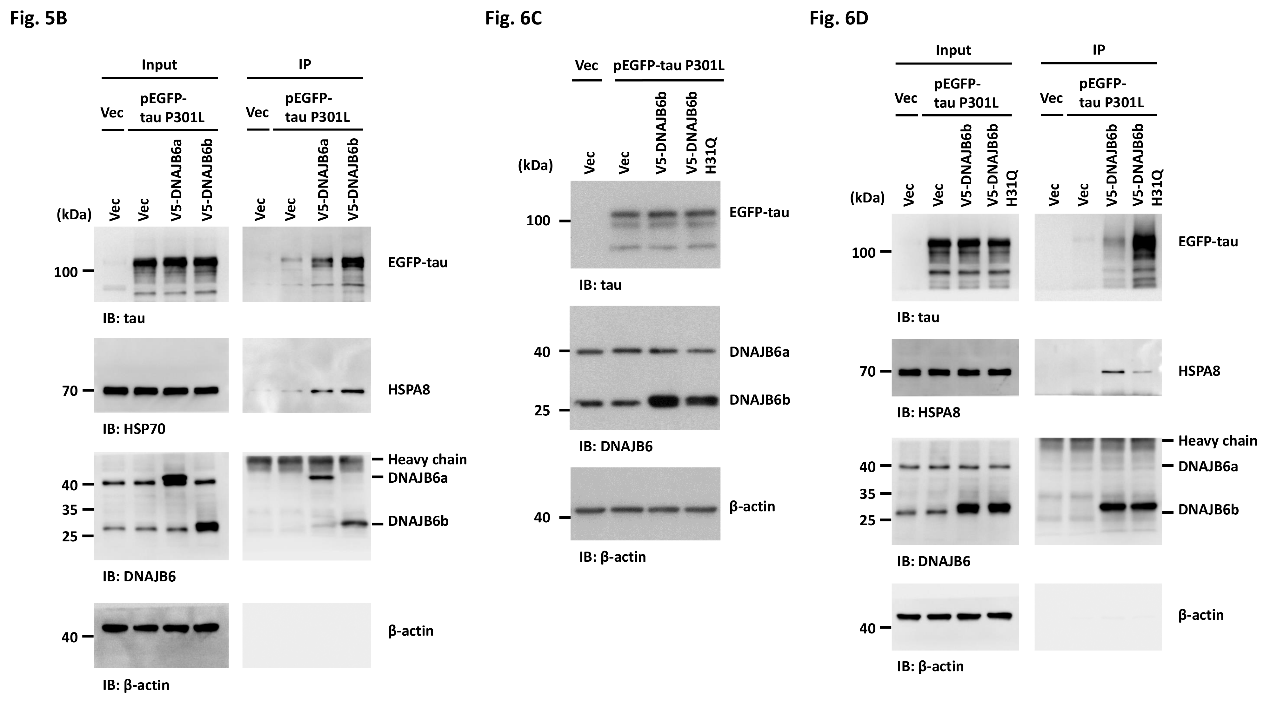
**

**
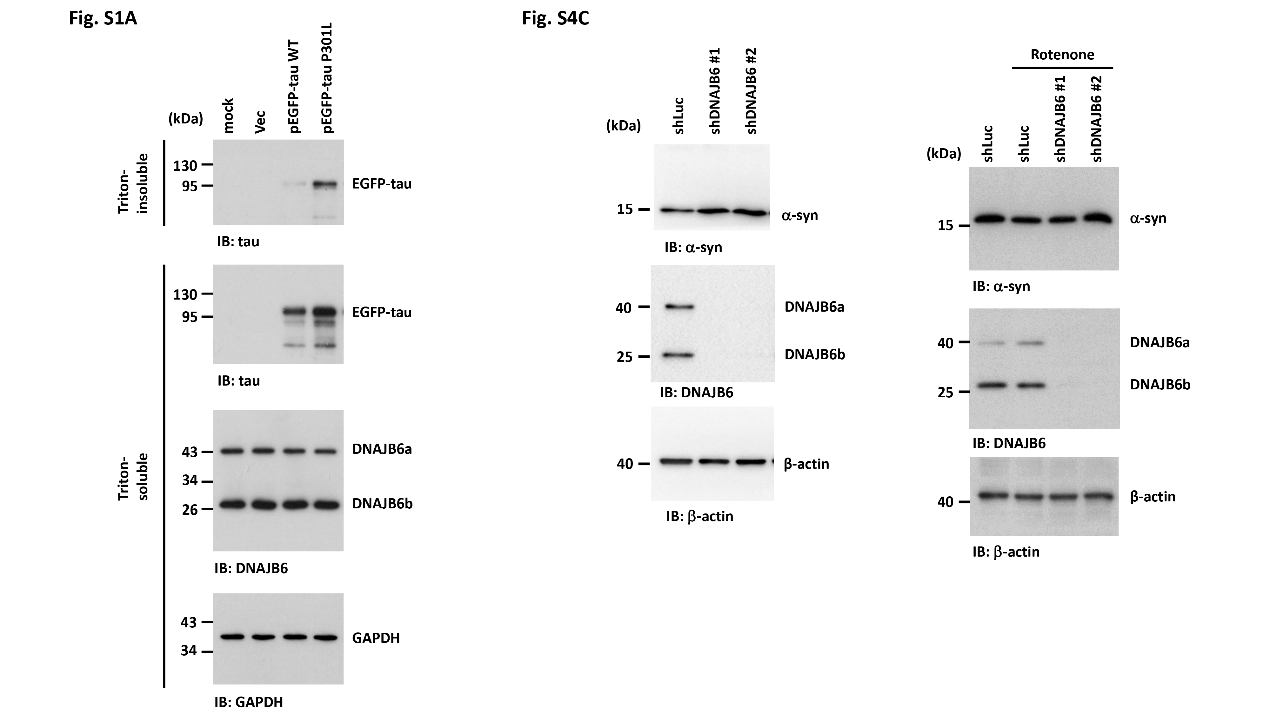
**

**
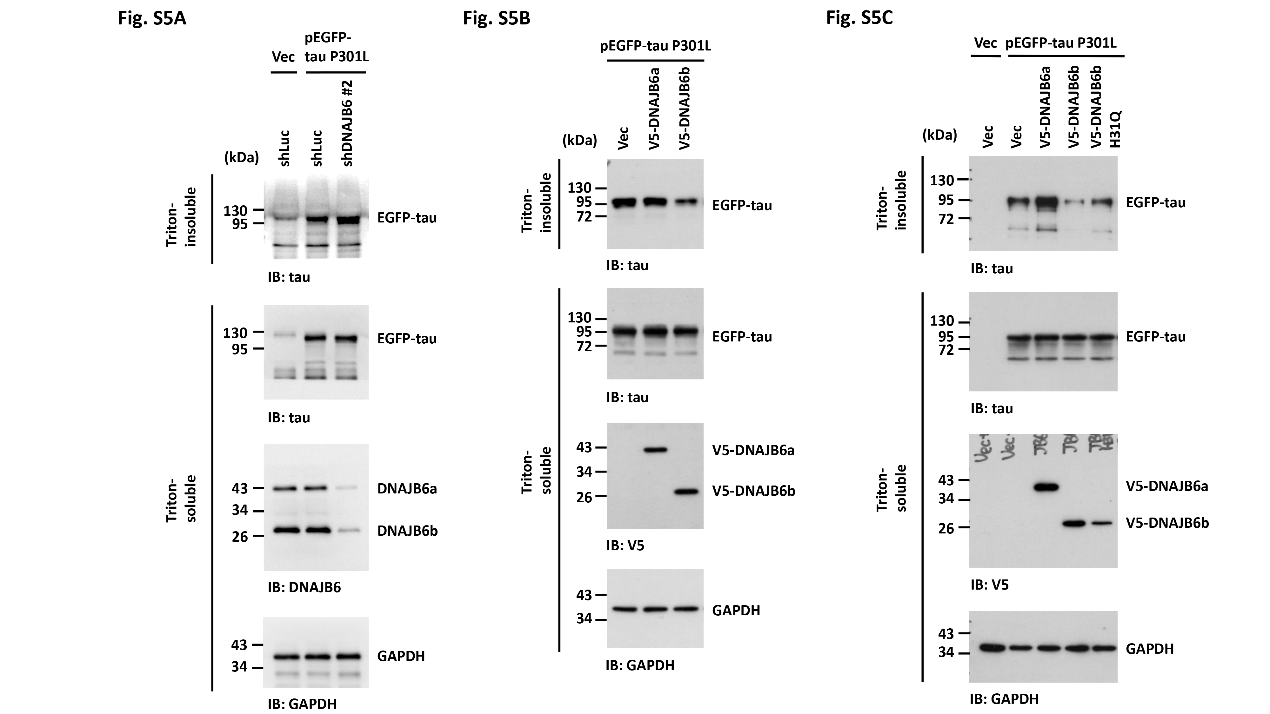
**

**
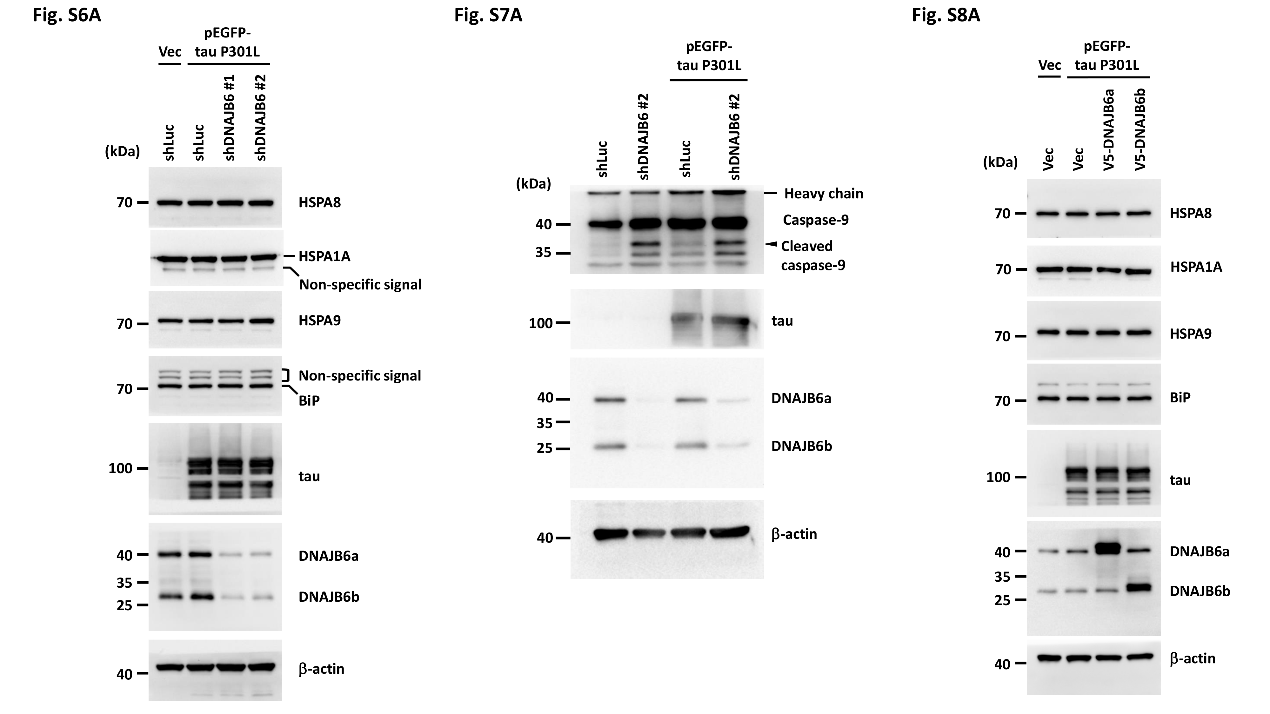
**

**Figure S10. Images of the full immunoblots.**

**Table S1**

| Oligonucleotide sequences of shRNA | Source | Identifier |
| --- | --- | --- |
| pLKO.1-shLuc  Target seq.: 5’GCGGTTGCCAAGAGGTTCCAT3’ | Academia Sinica | TRCN0000072249 |
| pLKO.5-shLuc  Target seq.: 5’ GCGGTTGCCAAGAGGTTCCAT3’ | Academia Sinica | TRCN0000231719 |
| pLKO.5-shDNAJA1 #1  Target seq.: 5’ CCAAGTAGAACTGGTGGACTT 3’ | Academia Sinica | TRCN0000275846 |
| pLKO.5-shDNAJA1 #2  Target seq.: 5’ GATATCAAGTGTGTACTAAAT3’ | Academia Sinica | TRCN0000275847 |
| pLKO.5-shDNAJA2 #1  Target seq.: 5’ TGATTTGCACATGACATATAA 3’ | Academia Sinica | TRCN0000293403 |
| pLKO.5-shDNAJA2 #2  Target seq.: 5’ GCCTGATTTGTTCTCAGCAAT 3’ | Academia Sinica | TRCN0000008785 |
| pLKO.1-shDNAJA3 #1  Target seq.: 5’ GCGAGTTCTCATCCTCTTCAT 3’ | Academia Sinica | TRCN0000008775 |
| pLKO.1-shDNAJA3 #2  Target seq.: 5’ TCCGACCTCTTTATTTCTATA3’ | Academia Sinica | TRCN0000008776 |
| pLKO.1-shDNAJB1 #1  Target seq.: 5’ CGGCTGTACCAAGAAGATGAA3’ | Academia Sinica | TRCN0000008791 |
| pLKO.1-shDNAJB1 #2  Target seq.: 5’ CGACGGAAAGAGCATTCGAAA3’ | Academia Sinica | TRCN0000008793 |
| pLKO.5-shDNAJB5 #1  Target seq.: 5’ ACTAGCTTTCAATCCAGTTTC3’ | Academia Sinica | TRCN0000436152 |
| pLKO.5-shDNAJB5 #2  Target seq.: 5’ GTCCACAGTGTCCAAGGTAAT 3’ | Academia Sinica | TRCN0000432799 |
| pLKO.1-shDNAJB6 #1  Target seq.: 5’ CGTTCAGTGGATTTCCGTCTT 3’ | Academia Sinica | TRCN0000008779 |
| pLKO.1-shDNAJB6 #2  Target seq.: 5’ GCCTCACCCGAGGATATTAAA3’ | Academia Sinica | TRCN0000008781 |
| pLKO.1-shDNAJC5 #1  Target seq.: 5’ CGCCAACCTTAGAATCATGAA3’ | Academia Sinica | TRCN0000154238 |
| pLKO.1-shDNAJC5 #2  Target seq.: 5’ GAAGCTTGCCTTGAAATATCA3’ | Academia Sinica | TRCN0000150949 |
| pLKO.5-shDNAJC6 #1  Target seq.: 5’ TTGTCTGAGAATAGGATTAAT 3’ | Academia Sinica | TRCN0000272478 |
| pLKO.1-shDNAJC6 #2  Target seq.: 5’ GCCTCTCACAATTAAGTCGAT 3’ | Academia Sinica | TRCN0000001903 |
| pLKO.1-shDNAJC11 #1  Target seq.: 5’ CCAGCACTGGTATCTGAGTAA 3’ | Academia Sinica | TRCN0000180192 |
| pLKO.1-shDNAJC11 #2  Target seq.: 5’ GTTTGGAAATTAGTCGTCTTT 3’ | Academia Sinica | TRCN0000183440 |
| pLKO.5-shDNAJC13 #1  Target seq.: 5’ CGTGATCCGGCAACCTATAAT3’ | Academia Sinica | TRCN0000243949 |
| pLKO.5-shDNAJC13 #2  Target seq.: 5’ GATCCTAATTGTATCACATTA3’ | Academia Sinica | TRCN0000243950 |
| pLKO.5-shDNAJC29 #1  Target seq.: 5’ GATCCTCTTGGATGCGTTATT 3’ | Academia Sinica | TRCN0000303351 |
| pLKO.5-shDNAJC29 #2  Target seq.: 5’ ATGTGTTGTAGCACGAATAAA 3’ | Academia Sinica | TRCN0000369556 |

**Table S2**

| Primers | Source | Sequence |
| --- | --- | --- |
| tau-XhoI-For | IDT | 5’TCAGATCTCGAGCTATGGCTGAGCCCCGCCAGGAGTTCGAAGTGATGGAAGATCACGCTG3’ |
| tau-KpnI-Rev | IDT | 5’GCCCGCGGTACCTCACAAACCCTGCTTGGCCAGGGAGGCAGACACCTCGTCAGCTAGC3’ |
| tau-P301L-AvrII-For | IDT | 5’GATAATATCAAACACGTCCTAGGAGGCGGCAGTGTGCAAA3’ |
| DNAJB6-H31Q-AflII-For | IDT | 5’AAAGGCATATCGGAAACTGGCACTTAAGTGGCAACCAGATAAAAATC3’ |

**Table S3**

| Plasmids | Sources | Identifier |
| --- | --- | --- |
| pEGFP-C1 | Clontech | Cat# 6084-1 |
| pMX-neo-tau (2N4R) | Hatakeyama et al. [36] | N/A |
| pEGFP-C1-tau (2N4R) | This study | N/A |
| pEGFP-C1-tau P301L (2N4R) | This study | N/A |
| VN-tau (P301L) | Addgene | Cat# 87634 |
| tau (P301L)-VC | Addgene | Cat# 87633 |
| pcDNA5/FRT/TO-V5 control | This study | N/A |
| pcDNA5/FRT/TO-V5-DNAJB6a | Addgene | Cat #19529 |
| pcDNA/FRT/TO-V5-DNAJB6b | Addgene | Cat #19528 |
| pcDNA5/FRT/TO-V5-DNAJB6b H31Q | This study | N/A |
| pCMV-Δ8.91 | Academia Sinica | N/A |
| pMD.G | Academia Sinica | N/A |

**Table S4**

| Oligonucleotides | Source | Sequence |
| --- | --- | --- |
| DNAJA1_RT-qPCR_Forward | IDT | 5’GGTGAAGGAGACCAAGAACCAG3’ |
| DNAJA1_RT-qPCR_Reverse | IDT | 5’AGCCACACAGTGCTTCAACGAG3’ |
| DNAJA2_RT-qPCR_Forward | IDT | 5’TTTCTTTCCACATTTGCCTG3’ |
| DNAJA2_RT-qPCR_Reverse | IDT | 5’ATAGACCAACAGATGTCCCT3’ |
| DNAJA3_RT-qPCR_Forward | IDT | 5’AGAAATGAAGCCTTAAGCTG3’ |
| DNAJA3_RT-qPCR_Reverse | IDT | 5’GAAGAACTCATTCTGGAACCT3’ |
| DNAJB1_RT-qPCR_Forward | IDT | 5’AGTTCAAGGAGATCGCTGAGGC3’ |
| DNAJB1_RT-qPCR_Reverse | IDT | 5’GCTGAAAGAGGTACCATTGGCAC3’ |
| DNAJB5_RT-qPCR_Forward | IDT | 5’GTGCTCTACAGTGCCCTGATCA3’ |
| DNAJB5_RT-qPCR_Reverse | IDT | 5’TCTTCACGGTGCCTGGCTTGAT3’ |
| DNAJB6_RT-qPCR_Forward | IDT | 5’CTCTGCGTTCAGTGGATTTCCG3’ |
| DNAJB6_RT-qPCR_Reverse | IDT | 5’TGAAGTTGCCCATGCCACTACC3’ |
| DNAJC5_RT-qPCR_Forward | IDT | 5’GGACAAGAACGCAACCTCAGATG3’ |
| DNAJC5_RT-qPCR_Reverse | IDT | 5’TTTTGTGGCGTCCGTGAGGATG3’ |
| DNAJC6_RT-qPCR_Forward | IDT | 5’CTGGACAGTCAGGAGTGGAAGA3’ |
| DNAJC6_RT-qPCR_Reverse | IDT | 5’GGTGCTCCAAATGGGTCAAAGG3’ |
| DNAJC11_RT-qPCR_Forward | IDT | 5’CCGAGACACTAAAACCAGCCAC3’ |
| DNAJC11_RT-qPCR_Reverse | IDT | 5’CACGAGTCTGATCGTCATCTTGG3’ |
| DNAJC13_RT-qPCR_Forward | IDT | 5’GGTTCCAATGTGCTTCCTGTTGC3’ |
| DNAJC13_RT-qPCR_Reverse | IDT | 5’GCTTCAGGTAGAATGTGCCCAAG3’ |
| DNAJC29_RT-qPCR_Forward | IDT | 5’CCTTCTCCTCATCTGTATCAGAC3’ |
| DNAJC29_RT-qPCR_Reverse | IDT | 5’AGCAGCCACAAGGTGAGGTTTC3’ |
| hRPL_RT-qPCR_Forward | IDT | 5’CAAGGCAAAGCGAAATTGGT3’ |
| hRPL_RT-qPCR_Reverse | IDT | 5’GCCCGTTCAGTCTCTTCGATT3’ |

**Table S5**

| Antibodies | Source | Identifier |
| --- | --- | --- |
| tau | GeneTex | Cat# GTX59570  RRID: AB_10731242 |
| tau | GeneTex | Cat# GTX112981  RRID: AB_10730753 |
| DNAJB6 | Abcam | Cat# ab198995  RRID: AB_2924896 |
| HSPA8 | Novus | Cat# NB120-2788  RRID: AB_2120309 |
| HSPA9 | Thermo Fisher Scientific | Cat# MA3-028  RRID: AB_325474 |
| BiP | Abcam | Cat# ab32618  RRID: AB_732737 |
| HSPA1A | Santa Cruz | Cat# sc-66048  RRID: AB_832518 |
| β-actin | Proteintech | Cat# 60008-1-Ig  RRID: AB_2289225 |
| V5 | Thermo Fisher Scientific | Cat# R960-25  RRID: AB_2556564 |
| α-synuclein | GeneTex | Cat# GTX112799  RRID: AB_10618470 |
| DNAJA3 | Santa Cruz | Cat# sc-18820  RRID:AB_668801 |
| AT8 | Thermo Fisher Scientifics | Cat# MN1020  RRID: AB_223647 |
| GST | Santa Cruz | Cat# sc-459  RRID: AB_631586 |
| Alexa Fluor 647 goat anti-rabbit | Jackson ImmunoResearch | Cat# 111-605-003  RRID: AB_2338072 |
| Alexa Fluor 647 goat anti-rabbit | Thermo Fisher Scientifics | Cat# A32733  RRID: AB_2633282 |
| Alexa Fluor 488 goat anti-mouse | Thermo Fisher Scientifics | Cat# A11001  RRID: AB_2534069 |

**Table S6**

| Chemicals and Reagents | Source | Catalog number |
| --- | --- | --- |
| DMEM | Cytiva | SH30022.02 |
| DMEM/F12 | Cytiva | SH30023.02 |
| Rotenone | Sigma-Aldrich | R8875 |
| Polybrene | Millipore | TR-1003 |
| Puromycin | Sigma-Aldrich | Cat# P8833 |
| Complete EDTA-free Protease Inhibitor Cocktail | Roche | Cat# 11836170001 |
| Protein G Mag Sepharose Xtra magnetic beads | Cytiva | 28967070 |
| Luminata^TM^ Crescendo Western HRP Substrate | Millipore | Cat# WBLUR0500 |
| TRIzol | Thermo Fisher Scientifics | Cat# 15596018 |
| DAPI | Thermo Fisher Scientifics | Cat# D1306 |
| Autofluorescence Eliminator Reagent | Millipore | Cat# 2160 |
| ProLong Gold Antifade Reagent | Thermo Fisher Scientifics | Cat# P36934 |
| Fluoromount^TM^ Aqueous Mounting Medium | Sigma-Aldrich | Cat# F4680 |

**Table S7**

| Critical commercial assays |  |  |
| --- | --- | --- |
| T-Pro non-liposome transfection reagent II | T-Pro Biotechnology | Cat# JT97-N002M |
| Lipofectamine^TM^ LTX reagent with PLUS^TM^ reagent | Thermo Fisher Scientific | Cat# 15338100 |
| Bio-Rad Protein Assay | Bio-Rad | Car# 5000006 |
| Maxima First Strand cDNA Synthesis Kit for RT-qPCR | Thermo Fisher Scientific | Cat# K1642 |
| KAPA SYBR FAST | KAPA Biosystems | Cat# KK4600 |
| KAPA HiFi PCR Kit | KAPA Biosystems | Cat# KK2101 |
| Duolink® In Situ Wash Buffers, Fluorescence | Merck | Cat# DUO82049 |
| Duolink® In Situ PLA® Probe Anti-Mouse PLUS | Merck | Cat# DUO92001 |
| Duolink® In Situ PLA® Probe Anti-Rabbit MINUS | Merck | Cat# DUO92005 |
| Duolink® In Situ Detection Reagents Red | Merck | Cat# DUO92008 |

**Table S8**

| Software | Source | Identifier |
| --- | --- | --- |
| Zen Blue2.6 | Zeiss | N/A |
| ImageJ | National Institutes of Health | https://imagej.nih.gov/ij/ |
| BioRender | N/A | https://biorender.com/ |

**Table S9**

| Other materials and instruments | Source | Identifier |
| --- | --- | --- |
| ChemiDoc™ Imaging System | Bio-Rad | Cat# 12003153 |
| 96-well dot-blot apparatus | Bio-Rad | Cat# 170-6545 |
| Polyvinylidene difluoride membrane | Millipore | IPVH85R |
| 0.2 μm cellulose acetate membranes | Sterlitech | CA023001 |
| ApoTome.2 microscope | Zeiss | N/A |
| Zeiss Imager.M2 fluorescence microscope | Zeiss | N/A |
| Zeiss LSM 710 confocal laser scanning system | Zeiss | N/A |
